# Multilayer Ti–Cu Oxide Coatings on Ti6Al4V: Balancing Antibacterial Activity, Mechanical Strength, Corrosion Resistance, and Cytocompatibility

**DOI:** 10.3390/jfb17010016

**Published:** 2025-12-26

**Authors:** Stefan Valkov, Maria P. Nikolova, Tanya V. Dimitrova, Maria Elena Stancheva, Dimitar Dechev, Nikolay Ivanov, Yordan Handzhiyski, Andreana Andreeva, Maria Ormanova, Angel Anchev, Margarita D. Apostolova

**Affiliations:** 1 Institute of Electronics, Bulgarian Academy of Sciences, 72 Tzarigradsko Chaussee Blvd., 1784 Sofia, Bulgaria; 2 Department of Mathematics, Informatics and Natural Sciences, Technical University of Gabrovo, 4 H. Dimitar Str., 5300 Gabrovo, Bulgaria; 3 Department of Material Science and Technology, University of Ruse “Angel Kanchev”, 8 Studentska Str., 7017 Ruse, Bulgaria; 4 Roumen Tsanev Institute of Molecular Biology, Bulgarian Academy of Sciences, Acad. Georgi Bonchev Str., Bl. 21, 1113 Sofia, Bulgaria; 5 Faculty of Physics, Sofia University “St. Kliment Ohridski”, 5 James Bourchier Blvd, 1164 Sofia, Bulgaria; 6 Department of Material Science and Mechanics of Materials, Technical University of Gabrovo, 4 H. Dimitar Str., 5300 Gabrovo, Bulgaria; anchev@tugab.bg

**Keywords:** Ti6Al4V, PVD coatings, magnetron sputtering, CuO–TiO_2_ thin films, multilayer coatings, surface engineering, electrochemical behaviour, antibacterial properties, cytocompatibility, metallothionein

## Abstract

Titanium alloys are widely used for biomedical implants, but their performance is limited by wear, corrosion, and susceptibility to bacterial colonisation. To overcome these drawbacks, multilayer Ti–Cu oxide coatings were deposited on Ti6Al4V substrates using direct current magnetron sputtering. Two multilayer architectures (6 × 2 and 12 × 2 TiO_2_/CuO bilayers) were fabricated and evaluated for their structural, mechanical, electrochemical, and biological properties. SEM/EDS and XRD confirmed well-adhered crystalline coatings consisting of rutile/anatase TiO_2_ and monoclinic CuO with uniform elemental distribution. The coatings increased surface roughness, improved adhesion, and enhanced hardness by up to ~180% compared to uncoated Ti6Al4V alloy. Compared to the bare substrate, electrochemical testing in simulated body fluid showed higher corrosion resistance of both coated samples, but particularly for the 12 × 2 multilayers. Both architectures provided sustained Cu^2+^ release over seven days without a burst effect. In vitro biological testing showed that both multilayer coatings achieved over 96% inhibition of Gram-positive bacteria such as *Staphylococcus aureus* and *Bacillus subtilis*, while exhibiting moderate antibacterial effects against Gram-negative strains (*Escherichia coli*, *Pseudomonas aeruginosa*). Despite the presence of copper, MG-63 osteoblast-like cells demonstrated sustained viability and successful extracellular matrix mineralisation, indicating excellent cytocompatibility of the coatings with bone-forming cells. These results demonstrate that multilayer Ti–Cu oxide coatings can effectively balance antibacterial performance, corrosion resistance, mechanical strength, and support bone cell integration, making them a promising strategy for the surface modification of titanium-based biomedical implants.

## 1. Introduction

One of the most widely used materials for bone-substitute implants, titanium (Ti) and its alloys, often provoke adverse biological responses, including poor osseointegration and early implant-related infections under complex physiological conditions. These complications frequently lead to surrounding bone loss and reduced implant lifespan. For example, the typical frequency of peri-implantitis in dental implants has been reported at 22%, with longer implantation time positively correlated with its occurrence [1]. In orthopaedic replacements, the risk of infection varies between 0.4% and 16.1%, depending on the severity of the fracture [2]. Most infections, commonly caused by Gram-positive bacteria such as *Staphylococcus aureus* and *Staphylococcus epidermidis*, can develop resistant biofilms that are difficult to eradicate with conventional treatments [3]. Consequently, once a periprosthetic infection occurs, a second surgery to substitute the implant is often required.

In addition, Ti-based alloys inevitably release non-compatible metal ions into bodily fluids, which may trigger harmful or allergic reactions [4]. Their relatively low wear resistance in load-bearing applications also generates wear debris that can accumulate in adjacent tissue, leading to bone resorption, implant loosening, and eventual failure [5]. Therefore, to achieve the necessary biocompatibility, antibacterial performance, and corrosion resistance, yet still keeping titanium’s outstanding bulk performance, it is essential to apply appropriate surface modifications to titanium-based materials.

Due to their excellent biocompatibility, TiO_2_ films have attracted increasing attention as biomedical coatings [6]. When uniformly doped with antimicrobial metallic elements, such films can effectively protect implant surfaces from microbial colonisation while maintaining high biocompatibility owing to the presence of TiO_2_. Among various ions investigated, in vitro studies have shown that copper ions exhibit stronger antibacterial activity than silver, zinc, cobalt, aluminium, or mercury, while retaining good biocompatibility [7,8,9]. Consequently, copper has been widely incorporated into biomedical materials to enhance their antibacterial potential [10].

Cu-doped TiO_2_ coatings and composites remain an active area of research, with numerous studies reporting improved antibacterial performance [11,12,13]. The biocidal effect of copper is primarily attributed to the release of Cu^+^ and Cu^2+^ ions, as demonstrated in Ti–Cu materials [14,15]. Oxidation of Ti–Cu, leading to the formation of CuO/Cu, has also been shown to effectively destroy bacteria upon direct surface contact [16]. Recent investigations further confirm that titanium alloys containing copper exhibit strong antibacterial activity against *Pseudomonas aeruginosa* (*P. aeruginosa*), *Escherichia coli* (*E. coli*), and *Staphylococcus aureus* (*S. aureus*) [17,18]. Beyond antibacterial properties, copper is an essential trace element for humans, playing a vital role in protein and enzyme synthesis necessary for survival [19]. Trace amounts of copper have been reported to enhance and upregulate osteoblastic activity and proliferation [20] by participating in enzyme-driven bone metabolic processes, including collagen and elastin cross-linking [21]. Concentrations ranging from 0.06 to 63.5 g/L have been shown to significantly stimulate the growth and development of mouse osteoblasts [22]. However, excessive copper can be harmful, as its ability to alternate between oxidation states enables electron transfer reactions that damage DNA and may cause genotoxic effects [23]. Therefore, introducing an optimal amount of copper into biomaterials is of great importance, as it combines potent antibacterial activity with the ability to support eukaryotic cell adhesion and proliferation, thus promoting tissue regeneration.

Developing nanostructured films for surface engineering is increasingly critical for achieving antibacterial performance, biocompatibility, and superior hardness or wear resistance. Physical vapour deposition (PVD) enables precise control over the nanostructure of materials during growth, leading to improved hardness and wear resistance [24]. Magnetron co-sputtering further allows fine control of component concentrations, enabling the fabrication of multicomponent composite layers [25].

In this context, sputtered Ti–Cu coatings with multilayer architectures can provide an optimal balance between mechanical strength, biocompatibility, and antibacterial properties. Stranak et al. specifically demonstrated that a thin Ti–Cu film deposited on implants can create a localised antibacterial environment while being relatively safe for osteoblast cells [26]. Their coating consisted of two distinct layers produced under different sputtering conditions: the topmost layer, deposited at increased pressure, contained predominantly Cu (90%) with <1% Ti, while the lower layer included 55% copper and 25% titanium. Importantly, the structure of the deposited film influenced the release of copper ions into the culture medium. Another study employed high-power pulsed magnetron sputtering (HiPIMS) to deposit TiCuO coatings containing 20%, 40%, and 80% Cu on Ti6Al4V alloy discs [27]. Coatings with 80 wt% Cu showed the highest antibacterial effectiveness, significantly reducing *S. epidermidis* biofilm density and planktonic cell populations, while maintaining negligible cytotoxicity toward human osteoblasts. These findings highlight TiCuO systems as promising candidates for implant coatings that combine antimicrobial activity with high surface hardness [28]. Moreover, titanium–copper oxide coatings have the added advantage of providing corrosion resistance to the substrate, even while releasing antibacterial copper ions [29].

The antibacterial efficacy and biocompatibility of such coatings, however, are highly dependent on film thickness and deposition conditions. In our earlier work, we demonstrated that single-layered Cu–O magnetron-sputtered coatings on commercially pure titanium (cpTi) exhibited thickness-dependent antibacterial effects against *E. coli*, reducing bacterial viability by up to 79.6% [30]. Yet, while thicker coatings (125 nm deposited for 15 min) provided greater antibacterial action, they also induced cytotoxic effects in MG-63 cells, lowering cell viability by as much as 60% relative to uncoated cpTi. This result indicated a possible balance between antibacterial effectiveness and cell compatibility. To address this challenge, in the present study, we developed two-component Ti–Cu oxide multilayer films on Ti6Al4V substrates using DC magnetron sputtering, to achieve an optimal balance between antibacterial effectiveness, mechanical and corrosion stability and biocompatibility. We address the cytotoxicity observed in our previous single-layer Cu–O coatings by reducing the Cu deposition time to 10 s and implementing a multilayer TiO_2_/CuO structure. This architecture lowers the overall copper load and enables more controlled ion release by embedding thin CuO layers between TiO_2_ layers, thereby mitigating cytotoxicity while maintaining antibacterial activity, even under potential surface wear. As already mentioned, the Ti-Cu oxide composite structure has great potential for application in modern biomedicine and implant manufacturing due to its antibacterial properties and negligible cytotoxicity toward human osteoblasts. However, a multilayered structure containing Ti-O and Cu-O-based layers for modern biomedical applications and implant manufacturing is currently less well investigated. Moreover, the influence of the thickness of the discussed multilayered structures on the important structural and functional characteristics of Ti-Cu oxide thin films is still not yet studied. Therefore, by varying the number of deposited TiO_2_ and CuO layers, we aim to regulate both the copper concentration and the kinetics of Cu ion release, ensuring safe interaction with human cells while maintaining efficient microbial suppression. Here, we report the fabrication and characterisation of antibacterial multilayered (6 × 2 and 12 × 2) Ti–Cu oxide coatings produced by magnetron sputtering, designed to combine high surface hardness, wear and corrosion resistance, improved cell compatibility, and antimicrobial functionality.

## 2. Materials and Methods

### 2.1. Sample Preparation

**Figure 1:**
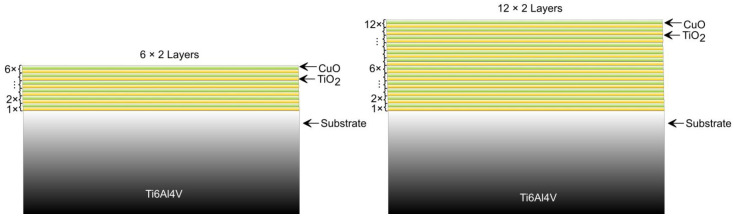
Schematic representation of the architecture of the designed 6 × 2 and 12 × 2 thin films. In the figure, green indicates CuO, whereas yellow corresponds to TiO_2_.

The composite Ti-Cu-O coatings were deposited by Direct Current (DC) magnetron sputtering, where 100 mm targets of Cu and Ti were used. After a large number of experiments, two multilayer architectures, comprising 6 and 12 TiO_2_/CuO bilayers, were applied onto a Ti6Al4V alloy substrate with dimensions of 14 × 14 mm and a thickness of 4 mm. These 6 and 12 multilayered films were considered the most representative and fully describe the effects of the layer number on the overall structure and the functional properties from a biomedical point of view. The substrate exhibited the following chemical composition in weight per cent: Al 6.12, V 4.00, Fe less than 0.40, O less than 0.20, H less than 0.015, N less than 0.05, C less than 0.08, with titanium constituting the balance. Before deposition, the substrate underwent fine grinding, followed by a 10-min sputter cleaning at a working pressure of 8 Pa and a discharge voltage of 950 V, and a discharge current of 0.1 A, in order to remove the residual contaminations. After that, it was heated to 360 °C. The deposition process of both kinds of layers was realised in an Ar-O_2_ atmosphere where the working pressure was 1.2 × 10^−2^ Pa. For the Cu-O-based layers, the discharge voltage was 370 V, and the discharge current was 0.5 A. The deposition time was 10 s. The Ti-O layers were deposited for 1 min per layer, with a discharge voltage of 400 V and a discharge current of 1 A. The deposition times correspond to thicknesses of about 15 nm for both kinds of layers. After the deposition of each layer (Cu-O or Ti-O), the target material was automatically changed to the other one to obtain 6 × 2 Cu-O/Ti-O and 12 × 2 Cu-O/Ti-O multilayered structures, respectively. During deposition, no negative bias voltage was applied to the substrate. The process parameters were optimised according to a number of experiments. Detailed information related to the deposition conditions, as well as to the film’s growth ratio, is published elsewhere [30,31]. Figure 1 presents the scheme of both kinds of coatings.

### 2.2. Samples’ Characterisation

The layers were characterised using a scanning electron microscope (SEM) system, KMAT EVO-10 (Zeiss, Oberkochen, Germany), equipped with an EDS microanalyser (Xplore 15, Oxford Instruments, High Wycombe, UK). During the measurements, the accelerating voltage was 20 kV, and the working distance was 8.9 mm. The EDS detector conducts true standardless analysis using P/B ZAF quantitative corrections.

X-ray diffraction analysis was performed to determine the phase composition of the deposited coatings (Empyrean X-ray diffractometer, Malvern Panalytical, Almelo, The Netherlands). The measurements were carried out in a symmetrical Bragg–Brentano mode using CuKα characteristic radiation (λ = 1.54 Å). Measurements were taken from 20° to 80° 2θ, with increments of 0.05° and 1-s counting time at each step.

MFP-3D Classic atomic force microscopy (AFM, Asylum Research, Oxford Instruments company, Abingdon, UK) was used to analyse the surface topography and morphology of the deposited coatings in non-contact mode, employing a probe with a tip radius of 10 nm. A scan area of 5 μm × 5 μm was selected along the x- and y-axes. Surface roughness was evaluated in six different regions for each sample. The average values of the arithmetic mean height (S_a_), the highest heights (S_z_) and the skewness (S_sk_) are presented in the results section below.

A CSEM-Macroscratch tester (CSEM, Neuchâtel, Switzerland) equipped with an optical microscope and a standard Rockwell-C diamond indenter (cone with a 200 μm tip radius and a 120° apex angle) was used to evaluate coating adherence up to a normal load of 30 N. The critical load F_c_ was determined using scratch track photos and an abrupt change in the coefficient of friction, which provides information about coating breakage. The coefficient of friction (COF) was determined using the macro scratch tester under an 8 N load, sliding at 0.3 mm/s for 30 s. Conducted at room temperature, each test was repeated three times, with the average friction values presented.

The DuraScan 20 Lite (ZwickRoell GmbH Co., KG, Ulm, Germany) tester was used to measure the microhardness at a peak force of 5 gf using a Knoop indenter and load control mode. To gather statistical information and evaluate data dispersion, ten measurements were performed on each polished and coated surface.

8.035 g of NaCl, 0.355 g of NaHCO_3_, 0.225 g of KCl, 0.176 g of K_2_HPO_4_ 3H_2_O, 0.311 g of MgCl_2_ 6H_2_O, 0.388 g of CaCl_2_, 0.072 g of Na_2_SO_4_, and 6.118 g of (CH_2_OH)_3_CNH_2_ (Tris) were all dissolved in 1 L of distilled water to prepare the simulated bodily fluid (SBF) solution. The solution was then stabilised at pH 7.4 using 1N HCl at 37 °C.

The sessile drop technique, combined with a camera-equipped contact angle device, was used to measure contact angles. Ethanol cleaned the surface before a 5.0 μL SBF droplet was placed from a microliter syringe. The droplet was brought into contact with the sample surface by moving the surface closer to the syringe tip. Five measurements per surface were taken at room temperature and 21% humidity, captured by a camera 30 s after droplet equilibrium. Contact angles were calculated from the images using Autodesk AutoCAD 2018. Together with the standard deviation values, mean contact angles were measured and recorded.

Electrochemical tests in the SBF solution were conducted using the Interface-1010E potentiostat (Gamry Instruments, Inc., Philadelphia, PA, USA). At 37 ± 0.5 degrees Celsius, the samples were submerged in 10 mL of naturally aerated SBF. The cell layout used three electrodes: the specimens as working electrodes (exposing 0.8 cm^2^), a saturated calomel reference electrode, and a platinum wire as a counter electrode. For the electrochemical investigations, the open circuit potential (OCP) stabilisation time was one hour. For potentiodynamic polarisation scans, the specimens were polarised to ±0.5 V_SCE_ against OCP, and a scan rate of 1 mV/s was employed. The samples were removed after the potentiodynamic polarisation test, and new samples were used to begin the electrochemical tests that followed.

After being submerged in SBF for one hour and seven days, the surface of coated samples and bare substrates was examined using electrochemical impedance spectroscopy (EIS). The EIS spectra were acquired using a ±5 mV RMS sinusoidal perturbation (versus OCP) and a frequency range of 10^4^ Hz to 10^−2^ Hz. The electrochemical data gathered from EIS were analysed using Gamry Echem Analyst 2 software (Gamry Instruments, Inc., Warminster, PA, USA). Most EIS measurements were performed in triplicate.

The release of copper ions was evaluated in 10 mL of SBF solution at 37 ± 0.5 °C. The concentration of copper ions in each sample solution was measured at predetermined immersion intervals using a spectrophotometer (HI83300, Hanna Instruments, Sat Nușfalău, Romania). A bicinchoninic acid assay was employed, forming a Cu–bicinchoninate complex with maximum absorbance at 562 nm, following an adapted EPA method, with a resolution of 0.001 mg/L and an accuracy of ±0.01 mg/L (±5%). Three coated samples of each type were tested in parallel, and the results are presented as the average of these measurements.

### 2.3. Biological Examinations

#### 2.3.1. Cell Culture

Human osteosarcoma MG-63 cells (CRL-1427, ATCC) were used in the experiments. The cells were maintained in high-glucose Dulbecco’s Modified Eagle Medium (DMEM + GlutaMAX, Gibco, Life Technologies Limited, Paisley, UK) supplemented with 10% foetal bovine serum (FBS Premium, Gibco), 100 U/mL penicillin, and 100 µg/mL streptomycin (Penicillin-Streptomycin-Glutamine 100×, Gibco). Cultures were incubated at 37 °C in a humidified atmosphere containing 5% CO_2_ until 80–90% confluence. MG-63 cells were routinely tested for mycoplasma contamination by DAPI staining (UltraCruz^®^ Aqueous Mounting Medium with DAPI, Santa Cruz Biotechnology, Dallas, TX, USA) and were found to be mycoplasma-free.

For all assays, 12-well culture plates were used. Samples from three independent technical experiments, each performed in triplicate per layer type (substrate, 6 × 2, and 12 × 2), were analysed. Before cell seeding, all sample surfaces were sterilised under a UV lamp for 30 min.

#### 2.3.2. Cell Attachment Assay

For the adhesion assay, MG-63 cells were seeded at 2.5 × 10^5^ cells/250 µL/sample and allowed to adhere for 3 h. Non-adherent cells were removed by three gentle washes with Phosphate-Buffered Saline (PBS). The remaining attached cells were detached with trypsin, centrifuged, and counted using an automated cell counter (Countess Automated Cell Counter, Waltham, MA, USA). The adhesion percentage was calculated by Equation (1):

Adhesion (%) = (Adhered cells for 3 h/Initially seeded cells) × 100(1)

#### 2.3.3. Cell Viability Assay

MG-63 cells were seeded on each sample at a density of 2.5 × 10^3^ cells/250 µL/sample and incubated for 24, 48, 72, 96, and 168 h. Cell viability was assessed using the AlamarBlue assay (AlamarBlue Viability Reagents, Invitrogen, Eugene, OR, USA). Before the addition of dye, the culture medium was replaced with FluoroBrite DMEM (FBD, Gibco). AlamarBlue reagent was added at a 1:10 (*v*/*v*) ratio (AlamarBlue: medium), and plates were incubated at 37 °C for 3 h. Fluorescence intensity was measured using a DTX 880 Multimode Detector (Beckman Coulter, Vienna, Austria) with excitation at 485 nm and emission at 535 nm under an area-scan protocol. Background fluorescence was determined from control wells containing AlamarBlue in FBD medium without cells and was subtracted from all measurements. Following each measurement, the dye solution was discarded, the wells were rinsed with PBS, and 1 mL of fresh growth medium was added prior to the next incubation.

#### 2.3.4. Immunofluorescence Staining

For immunofluorescence analysis, MG-63 cells were seeded at 1 × 10^5^ cells/sample in 200 µL of medium and incubated overnight at 37 °C. Samples underwent PBS washing followed by fixation in 3.7% methanol-free paraformaldehyde (from 16% stock, Pierce) in PBS for 15 min. F-actin filaments were stained with Alexa Fluor^®^ 488 Phalloidin (Invitrogen, Life Technologies, Carlsbad, CA, USA) for 2 h, followed by three PBS washes and two final rinses with distilled water. Samples were mounted using UltraCruz Mounting Medium with DAPI (Santa Cruz Biotechnology, Dallas, TX, USA). Fluorescence images were acquired with a Zeiss Axiovert 200 M microscope (Zeiss, Oberkochen, Germany).

Image processing and orientation analysis were performed in Fiji (ImageJ v.1.54p) [32] using the OrientationJ plugin [33]. F-actin micrographs were first converted to 8-bit greyscale and background-corrected using the rolling-ball algorithm (radius: 50 px). No edge-enhancing or Laplacian-of-Gaussian filters were applied to avoid artificial modification of filament gradients.

OrientationJ Analysis was run on the F-actin channel using cubic-spline gradient estimation and a window radius of 2 px. For each image, the plugin generated (i) an orientation map representing local fibre direction via a fixed hue–saturation colour LUT, and (ii) a coherency map expressing local alignment strength on a scale from 0 (isotropic) to 1 (perfectly aligned).

Ten non-overlapping regions of interest (ROIs) of identical size were manually selected within each image using the ROI Manager, avoiding cell-free areas, debris, or overlapping nuclei. OrientationJ measure was then applied to each ROI to extract coherency values, which served as the primary quantitative metric of cytoskeletal alignment. For each coating condition, coherency distributions were compiled across all images and ROIs. The Step-by-Step Protocol for OrientationJ Workflow is given in the Supplementary Materials.

Statistical comparisons between coating groups were performed on the ROI-level data using the Kruskal–Wallis test followed by Dunn’s post hoc correction (GraphPad Prism v.9 for Windows, GraphPad Software, San Diego, CA, USA, www.graphpad.com (accessed on 9 November 2025)). Violin plots were generated to visualise coherency distributions, with individual ROI values overlaid and median/interquartile range indicated for each group.

#### 2.3.5. Alizarin Red S Staining

Calcium deposition was evaluated using Alizarin Red S (ARS) staining (EMD Millipore Corp, Darmstadt, Germany). MG-63 cells were grown in growth medium for up to 21 days, followed by fixation with 4% methanol-free paraformaldehyde for 15 min at room temperature. After washing three times with distilled water, the cells were incubated with a 40 mM Alizarin Red S solution for 20 min at room temperature. Excess dye was removed by multiple washes with distilled water. Bound ARS was extracted using 10% acetic acid at room temperature, and absorbance was measured at 405 nm using a DU-50 spectrophotometer (Beckman Coulter, Brea, CA, USA).

#### 2.3.6. Western Blot Analysis

For protein analysis, 7 × 10^5^ cells/250 µL/sample were seeded onto each coated layer and substrate. After 24 h incubation, cells were lysed in buffer containing 50 mM HEPES (pH 7.7), 250 mM KCl, 10% glycerol, and 0.1% Nonidet P-40, supplemented with protease inhibitors (0.4 mM sodium vanadate, 0.5 mM PMSF, 1 mM DTT, 2 µg/mL leupeptin, and 2 µg/mL pepstatin). Lysates were incubated on ice for 30 min and stored overnight at –20 °C. Protein concentration was determined using the Pierce Detergent Compatible Bradford Assay Kit (Thermo Fisher Scientific, Rockford, lL, USA) following the manufacturer’s guidelines. Equal protein amounts were run on SDS-PAGE on 15% polyacrylamide gels and transferred to PVDF membranes. Membranes were blocked in 3% non-fat dry milk in PBS, then incubated for 1 h with primary antibody against metallothionein [34]. β-actin was used as a loading control and detected with β-actin antibody (sc-47778, Santa Cruz Biotechnology, Dallas, TX, USA) diluted in PBS containing 0.05% Tween-20. After washing, membranes were treated for 1 h with horseradish peroxidase (HRP)-linked secondary antibodies, including m-Igκ BP-HRP (sc-516102) or donkey anti-rabbit IgG-HRP (sc-2313), sourced from Santa Cruz Biotechnology. Immunoreactive bands were visualised using ECL Select Western Blotting Detection Reagent (Amersham) and recorded on X-ray film. The ECL signals were analysed with Fiji (ImageJ v.1.54p) [32].

#### 2.3.7. Antibacterial Evaluation

The antibacterial activity of the coatings was evaluated by the plate count method. Four bacterial strains were used: Escherichia coli K12 AB1157 (F^−^ thr-1 leu-6 proA2 his-4 argE3 thi-1 lacY1 galK2 ara-14 xyl-5 mtl-1 tsx-33 rspL31 supE44), Bacillus subtilis NCIB 3610, *Pseudomonas aeruginosa* NBIMCC 3700 (National Bank for Industrial Microorganisms and Cell Cultures, Sofia, Bulgaria), and *Staphylococcus aureus* ATCC 6538P (ATCC, Manassas, VA, USA). A single colony of each strain was inoculated into 5 mL of sterile Lysogeny Broth (LB) medium containing 1% protein hydrolysate, 0.5% yeast extract, and 0.5% NaCl (pH 7.4), and incubated overnight at 37 °C under constant agitation. The following day, 100 µL of the overnight culture was transferred into 10 mL of fresh sterile LB medium and cultivated at 37 °C until the optical density at 600 nm (OD_600_) reached 0.6. Subsequently, 1 mL of the bacterial suspension was centrifuged at 2300 rpm for 7 min, the supernatant discarded, and the pellet resuspended in 1 mL of sterile phosphate-buffered saline (PBS, pH 7.4). Square samples (1.4 × 1.4 cm^2^) of the tested coatings and the uncoated Ti6Al4V substrate were flame-sterilised for 3 s and placed in 12-well culture plates. Each sample was covered with 150 µL of bacterial suspension. After incubation for 24 h at 37 °C, 10 µL aliquots from each well were serially diluted: 10^−5^ for *E. coli*, *B. subtilis*, and *P. aeruginosa*, and 10^−4^ for *S. aureus*. Then, 100 µL of each dilution was spread on LB agar plates and incubated for 24 h at 37 °C. The resulting colonies were photographed and enumerated to determine colony-forming units (CFU). The antibacterial efficacy (R, %) was calculated using Equation (2):

R = (B − A)/B × 100, %(2)

where A and B denote the mean CFU counts for the test and control Ti6Al4V samples, respectively. All experiments were conducted in triplicate using three independent technical replicates per condition.

#### 2.3.8. Biofilm Formation

The Live/Dead L10316 FilmTracer Biofilm Viability Kit (Invitrogen Inc., Carlsbad, CA, USA) was used to analyse biofilm formation and the viability of adhered bacteria on the surfaces. This kit contains two stains: SYTO-9, which labels live cells in green, and propidium iodide (PI), which labels dead cells in red. The working solution of fluorescent stains was prepared according to the manufacturer’s instructions in sterile PBS.

Before staining, the bacterial suspension, prepared as described in Section 2.3.7, was grown on all surfaces for 24 h. Firstly, the samples were carefully rinsed with 1 mL of PBS three times for five minutes on an orbital shaker. Subsequently, 200 μL of staining solution was applied to each biofilm, which was then covered and protected from light, and incubated for 30 min at room temperature. The next step was to rinse the samples carefully with 1 mL of PBS three times for five minutes on a shaker, followed by one rinse with 1 mL of distilled water for each sample. Finally, using UltraCruz fluorescence mounting medium (Santa Cruz Biotechnology, Dallas, TX, USA), the samples were prepared and observed under a Zeiss Axiovert 200 M fluorescence microscope at 63× magnification with an oil-immersion lens (Zeiss, Oberkochen, Germany).

Image stacks were processed using Fiji (ImageJ v.1.54p) (NIH). Quantitative biofilm parameters were extracted as follows: live biomass (µm^2^), defined as the total projected area of pixels classified as live based on SYTO 9 fluorescence; dead biomass (µm^2^), defined as the projected area of pixels classified as non-viable based on propidium iodide fluorescence; and total biomass (µm^2^), calculated as the sum of live and dead biomass. Cell viability and inhibition of the live biofilm fraction were computed using the following Equations (3) and (4):

Cell viability (%) = (Live biomass/Total biomass) × 100(3)

Inhibition of live biofilm (%) = (1 − (Live biomass_sample/Live biomass_control)) × 100(4)

All measurements were obtained from three independent biological replicates, and five non-overlapping fields-of-view per replicate were analysed.

### 2.4. Statistical Analysis

All data are presented as mean ± standard deviation (SD) from at least three independent technical experiments, each performed in triplicate. Statistical analysis was conducted using ANOVA followed by Tukey’s post hoc test for multiple comparisons.

Fluorescence intensity values from the Alamar Blue assay (*n* = 9 per sample and time point) were analysed using IBM SPSS Statistics (PASW 18.0 IBM Corp., Armonk, NY, USA). Data were first tested for normality by the Shapiro–Wilk test and for homogeneity of variance by Levene’s test. Since the data met parametric assumptions, a mixed (split-plot) repeated-measures ANOVA was performed, with Time (24, 48, 72, 96, and 168 h) as the within-subjects factor and Sample (substrate, Coated 6 × 2, and Coated 12 × 2) as the between-subjects factor. Mauchly’s test of sphericity was used to assess the equality of variances of differences, and, where sphericity was violated, Greenhouse–Geisser corrections were applied. Polynomial trend analyses were conducted to evaluate linear, quadratic, and cubic components of the time-dependent response. Post hoc comparisons were performed with Bonferroni adjustment for multiple testing. Statistical significance was set at *p* < 0.05.

## 3. Results and Discussion

### 3.1. Structural Characterisation

**Figure 2:**
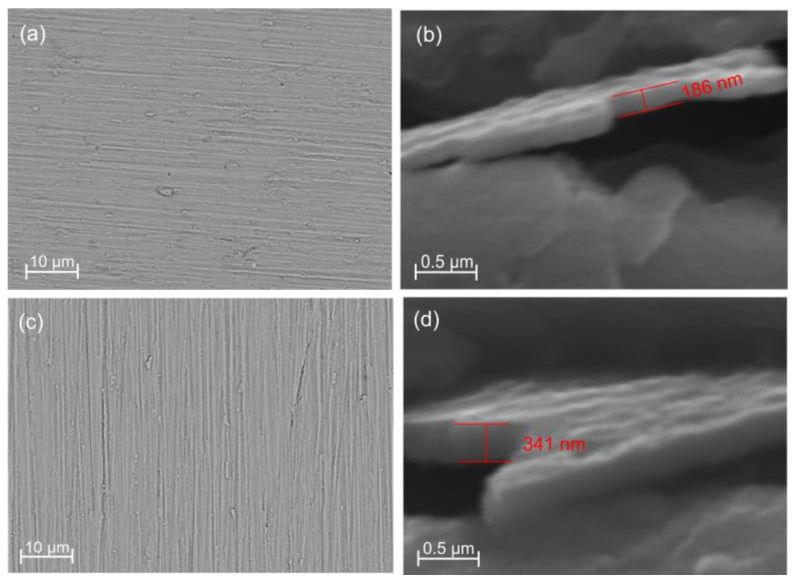
Representative SEM micrographs showing surface morphology (**left**) and cross-sectional thickness (**right**) of the (**a**,**b**) 6 × 2 and (**c**,**d**) 12 × 2 oxide coatings. The images highlight the uniform surface of the multilayers and the increase in total coating thickness with additional TiO_2_/CuO cycles.

Surface morphology for both 6 × 2 and 12 × 2 coatings is displayed in Figure 2a,c. Both coatings produced by DC-MS have a low number of surface flaws and an excellent superficial aspect. The average thickness of the 6 × 2 films is 178 ± 7 nm, and that of the 12 × 2 films is 335 ± 8 nm, respectively, according to SEM tests done on the cross-sections (Figure 2b,d), showing nearly a twofold difference in thickness. These measurements were done according to ten independent experiments at completely different regions of the samples. It could be clearly seen that the standard deviation in the measurements of the film’s thickness is less than 10 nm, highlighting the uniformity of the films deposited across the entire substrates in both considered cases. The layers have bonded to one another quite well, and there are no cavities or fissures visible, which could enhance the coating’s resistance to corrosion.

**Figure 3:**
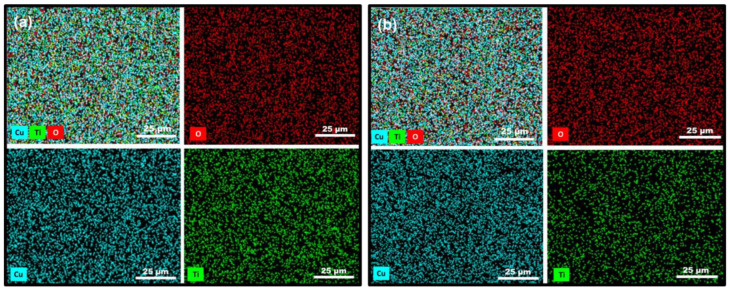
SEM-EDS elemental mapping images illustrating Ti, Cu, and O presence within the (**a**) 6 × 2 and (**b**) 12 × 2 coatings, demonstrating a uniform incorporation of the elements throughout the multilayered films.

Figure 3 shows the EDS elemental maps of the coated samples, illustrating the surface distribution of Ti, Cu, and O. The results reveal that all detected elements are uniformly distributed across the coatings, indicating a homogeneous film composition. As shown in Table 1, the copper and oxygen contents increase with increasing deposition time. For the 6 × 2 films, the copper concentration is around 30 wt%; for the 12 × 2 coatings, it is approximately 40 wt%. The small thickness of the films may affect the measured titanium content, as X-ray signals originating from the underlying substrate can contribute to the overall detected intensity.

**Figure 4:**
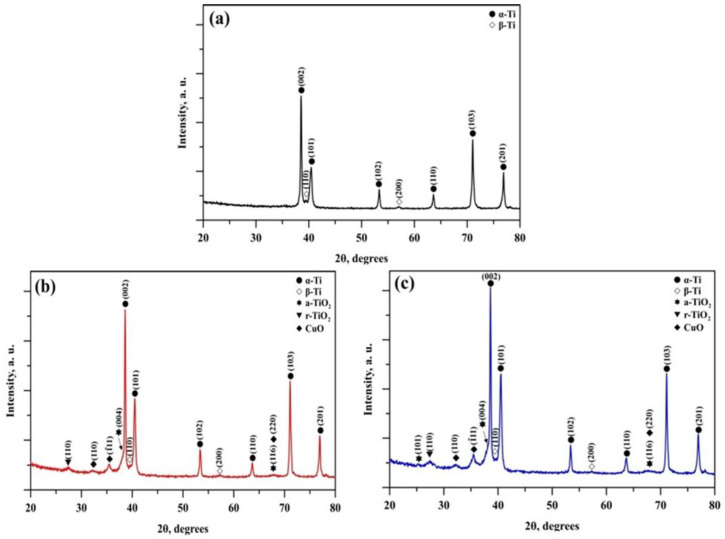
XRD pattern of the (**a**) bare, (**b**) 6 × 2 and (**c**) 12 × 2 coated samples, illustrating the crystalline phases present in the coatings. In addition to the α- and β-titanium phases originating from the substrate, the coated samples exhibit reflections corresponding to anatase (a-TiO_2_), rutile (r-TiO_2_), and CuO.

The experimentally obtained XRD patterns of the base Ti6Al4V substrate, as well as of the deposited Ti-Cu-O coatings, are presented in Figure 4. Figure 4a shows the diffractogram corresponding to the base material, Figure 4b shows the XRD pattern of the coating consisting of 6 × 2 layers, and Figure 4c presents the spectrum of the coating containing 12 Cu-O/Ti-O layers. The XRD results were evaluated following the International Centre for Diffraction Data (ICDD). The patterns exhibit a relatively low background. Amorphous-like halos were absent in all the cases considered. This confirms the absence of amorphous inclusions, meaning that the deposited films are characterised by a high degree of crystallinity. Upon evaluation of the phase composition of bare Ti6Al4V (Figure 4a), the experimentally obtained diffraction pattern exhibits peaks corresponding to α-titanium, having a hexagonal close-packed (HCP) crystal structure, as well as β-Ti, characterised by a body-centred cubic (BCC) structure. This phase composition is typical for the alloy used as a substrate material. It is important to note that the (110) peak position of beta-titanium is slightly upshifted compared to the data available in the ICDD database. Nonetheless, the peak intensity and position data in the referenced crystallographic database reflect the high-temperature phase characteristics. However, in the present particular case, the observation of the beta-structure inclusions within the Ti6Al4V substrate is due to the existence of the beta-stabilising vanadium element in the alloy. This means that the lattice parameters, and therefore, the beta phase diffraction peaks, may not precisely match the database records, as the vanadium element’s atomic radius affects their positions [35].

In the phase composition of the 6 × 2 layered film, in addition to the α and β phases, coming from the substrate, three more phases can be distinguished, namely TiO_2_ in the form of anatase and rutile (both with tetragonal crystal structure), as well as CuO, characterised by a monoclinic crystal structure. Thus, the deposited coating consists of a three-phase structure comprising anatase and rutile TiO_2_, along with CuO. Notably, in the experiment, the peak intensities measured for the TiO_2_ phase in the form of anatase are much less pronounced in comparison with those of the rutile structure, meaning that the content of the latter one is much lower. This result contradicts those published in Ref. [36]. However, the TiO_2_ coatings considered in the study [36] were deposited at 180 degrees Celsius, which is twice lower than the deposition temperature applied in the present case. Hence, the high-temperature deposition conditions are responsible for the substantial rutile phase observed in the coating. As already mentioned, the deposition process took place at 350 °C. In this case, the mobility of the sputtered particles on the top of the substrate (or on the top of the already deposited coating) is much higher than the case presented in Ref. [36], which could be considered as a prerequisite for the formation of the discussed structure. Also, the detected phase composition of the coating, consisting of 12 × 2 layers, does not differ from that of the thinner one (6 × 2 coated sample). This suggests that film thickness has no impact on phase composition in the examined range, corroborating the findings of [30]. However, it could be noted that the intensities of the experimentally obtained diffraction maxima corresponding to the Ti-Cu-O coating are stronger in the case of the 12 × 2 coated specimen, which is attributed to the higher thickness of the deposited film. It should be highlighted that the positions of the maxima peaks for both TiO_2_ and CuO phases do not differ significantly as a function of the number of applied TiO_2_ and CuO layers. This means that the increase in the number of deposited layers does not lead to the formation of additional residual stresses, variations in the lattice parameters and unit cell volumes of the discussed oxide-based phases. This could also have some potential practical benefits [37,38].

**Figure 5:**
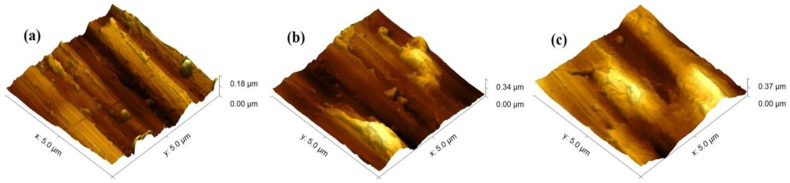
3D AFM images illustrating the surface structure of the samples: (**a**) bare substrate; (**b**) 6 × 2 and (**c**) 12 × 2 coated specimens. The coated surfaces exhibit increased roughness and the development of nanoscale morphological features associated with the sequential TiO_2_/CuO layer growth.

Figure 5 presents the surface topography analysis of both coated and uncoated samples, conducted using AFM. To better understand the surface architecture and topography of both the deposited coatings and the base substrate material, the mean roughness (S_a_), maximum height (S_z_), and Skewness (S_sk_) parameters are presented in Table 2. The sign of S_sk_ indicates the dominance of the valley structures (S_sk_ < 0) or the prevalence of surface peaks (S_sk_ > 0). In this sense, if the S_sk_ value is closer to zero, the distribution of the height of peaks to the depths of valleys is closer to symmetrical. Based on the results in Table 2, it is evident that coating deposition causes an increase in surface roughness (S_a_ parameter). The mean roughness of the uncovered and preliminary polished Ti6Al4V substrate is about 20 nm and increases to 45 nm after the deposition of the thinner (6 × 2 layered) coating. This is due to several contributing factors. The formation of the film starts with nucleation, which represents small islands on the surface of the substrate. Usually, these islands grow and coalesce. However, in most cases, they do not grow uniformly, creating bumps and voids, which could be considered a basic reason for the increase in the surface roughness. From another viewpoint, the fabricated coatings have a polycrystalline nature, meaning that the grains grow in different directions with different sizes, which could also be a reason for the increase in surface roughness. The shadowing effect of some residual surface formations on the substrate should also contribute to the observed higher roughness in the case of deposition of 6 × 2 films. The same can be said about the thicker coating (12 × 2 layers). The measured roughness in this case is about 50 nm, or similar to the thinner one. The authors of [39] report that increasing film thickness results in a reduction in surface roughness. However, in Ref. [39], the variations in thicknesses considered are much larger (span 450 nm) than in the present particular case (span 150 nm). Because of the insignificant thickness fluctuation in our work, the measured roughness should not be impacted much. These statements are also confirmed by the measured highest heights (S_z_ parameter) of the films. In both considered coatings, S_z_ is about 0.35 µm. However, an interesting trend can be observed for the S_sk_ parameter. In all cases, the value is positive, indicating that peak heights exceed valley depths. For the uncoated substrate, it is 0.079 and increases to more than 1. This again could be attributed to the nucleation and formation of islands. Nevertheless, in the case of 12 × 2 CuO/TiO_2_ layers, it decreases to 0.303, which could be attributed to the further growth of the film and filling of the valleys. The better sealing of surface discontinuities, such as valleys or defects, by thin PVD film layers has been shown to delay corrosive media penetration into underlying substrates, reduce corrosion vulnerability and improve coating effectiveness [40].

### 3.2. Mechanical Characteristics of the Coated Systems

Table 3 shows the microhardness values for both 6 × 2 and 12 × 2 coated specimens and the bare alloy. The surface hardness of the Ti6Al4V alloy was enhanced by roughly 65% and 180% after coating with 6 × 2 and 12 × 2 films, respectively. According to our research, the hardness of the coatings also increases as the number of Ti-Cu oxide film deposition layers increases. The highest indentation depth, however, is greater than 10% of the coating thickness, indicating that the substrate hardness affects the measured hardness values.

**Figure 6:**
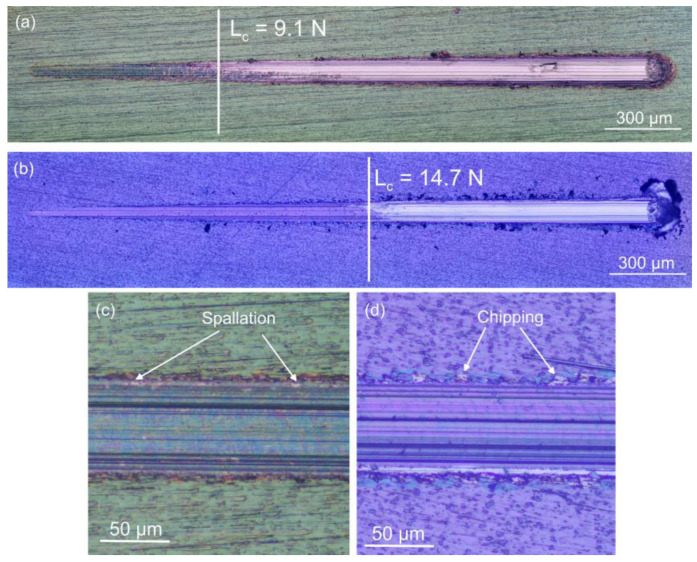
Representative scratch track images of the complex oxide films on Ti6Al4V alloy: (**a**) 6 × 2; (**b**) 12 × 2, showing the scratch path and the corresponding critical load (Fc) at which complete coating detachment occurs. Panels (**c**,**d**) present micrographs of the damage produced under a constant load of 10 N for the 6 × 2 and 12 × 2 coatings, respectively. Arrows indicate localised spallation and chipping sites observed along the scratch tracks.

Strong adhesion between the coating and substrate is essential to protect Ti implant materials from abrasion due to mechanical wear, as titanium and its alloys inherently have low wear resistance. Scratch testing was used to evaluate coatings’ resistance to loads under both normal and tangential forces. The results are presented in Figure 6 and Table 3. A significant change in the scratch track view and coefficient of friction highlighted the critical load (F_C_) brought on by coating deterioration. Following some buckling, the 6 × 2 coating fully detached from the substrate at an average critical load of about 9.2 N (Figure 6a). Figure 4b shows a scratch track result near the average F_c_ value (14.8 N) of the thicker films. After this loading, the substrate was fully exposed till the test finished. The higher F_c_ value implies a stronger link between the film and the substrate, allowing the coated system to sustain a greater load. This threshold effect occurs when the coating’s fracture toughness and adhesion limit are exceeded, leading to gradual failure rather than abrupt delamination, as seen in Figure 6a,b. The observed behaviour is primarily influenced by the difference in hardness between the coating and Ti6Al4V substrate, as well as the small thickness of the coatings, rather than by catastrophic interfacial adhesion failure. In both considered cases, it is well visible that the sublayers constructing the overall coating systems are well adhered to each other. Following the results presented in Figure 6, a detachment of the film was observed directly from the substrate. Up to that point, the coatings were destroyed evenly, pointing to the good adhesion between the sublayers in both cases. For comparison, the adhesion performance of our multilayer oxide films can be related to literature reports. Ding et al. [41] observed that amorphous Ta_2_O_5_/Ta_2_O_5_–Ti/Ti multilayers (~6.1 µm thickness) deposited on Ti-6Al-4V exhibited a critical load (F_C_) of 13.38 N. In contrast, Noori et al. [42] reported significantly higher F_C_ values for harder crystalline nitride coatings, with 39.6 N for CrN/TiN (3.08 µm) and 39.3 N for CrN/ZrN (2.8 µm) on the same substrate. While the critical loads measured for our multilayer oxide films are lower than those of the nitride coatings, the results demonstrate that even ultrathin oxide multilayers provide measurable mechanical protection, and that the multilayer architecture effectively enhances adhesion and durability relative to thicker multilayer Ta_2_O_5_ coatings. Therefore, the combination of a stiff, hard multilayer coating and a mechanically robust Ti6Al4V substrate provides both surface resistance to scratch-induced failure and adequate bulk mechanical support for implant applications.

Because of the enhanced hardness of oxide coatings, the films’ coefficient of friction (COF) at 10 N is lower than that of the bare substrate (Table 3). The optical micrographs of the wear tracks are shown in Figure 6c,d. For the 6 × 2 deposited oxide films, the COF drops to roughly 0.19, while it is equal to about 0.26 for the 12 × 2 coatings. The oxide-coated samples exhibit a coefficient of friction that is roughly 47% and 28% lower for the 6 × 2 and 12 × 2 deposited films, respectively, than the untreated sample. Because the Ti6Al4V alloy substrate has low hardness, the indenter penetrated it easily, causing plastic deformation on the surface. The 6 × 2 deposited film (Figure 6c) remains intact, probably because of higher plasticity, unlike the wear scar on the 12 × 2 coatings, where the indenter contact damages parts of the films, causing small spallations and some lateral chipping along the tracks (Figure 6d).

### 3.3. Wettability

**Figure 7:**
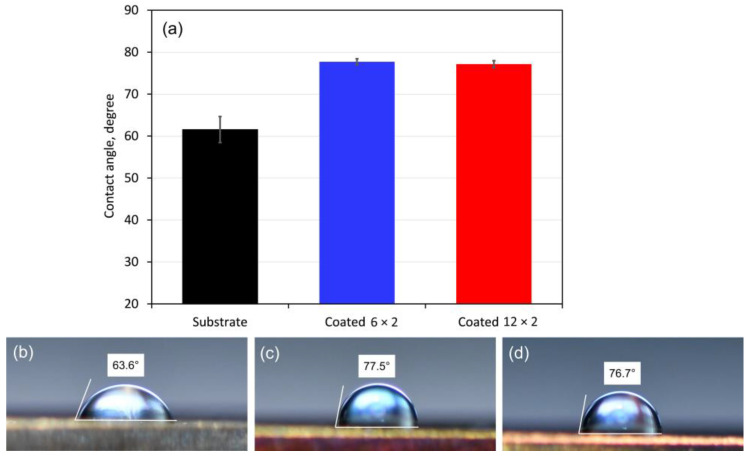
Average contact angle values (**a**) measured using 5 µL SBF droplets, along with representative side-view images of droplets on the surfaces of (**b**) the bare substrate, (**c**) the 6 × 2 coating, and (**d**) the 12 × 2 coating. Error bars represent the standard deviation calculated from multiple measurements.

Contact angle measurement methods are commonly used to determine surface wettability, and they typically demonstrate that a lower contact angle of hydrophilic surfaces is linked to a higher degree of bioactivity [43]. The coatings’ wettability was assessed by measuring their contact angles using drops of simulated body fluid (SBF), chosen for its ionic composition similar to human blood plasma. Figure 7 presents the contact angle values obtained and the typical shape of SBF droplets on each coated surface. The substrate and coating surfaces exhibit hydrophilicity, demonstrated by SBF contact angles below 90° on all samples. Both coated samples had a contact angle greater than 75 degrees in comparison to the control sample, suggesting a propensity for hydrophobic behaviour. However, contact angles below 90° indicate hydrophilicity, which is generally favourable for bone implants. Wilson et al. [44] showed that hydrophilic surfaces preserve fibronectin’s cell-adhesive function, enhancing cell response, while hydrophobic surfaces reduce it. Increased surface hydrophobicity also promotes adhesion of pathogens like *S. aureus* and *S. epidermidis* [45]. Adhesion and proliferation of human mesenchymal stem cells (hMSCs) and mouse MSCs were enhanced on hydrophilic surfaces with contact angles of 40–70° and 70–90°, respectively, while moderate wettability (20–70°) promoted spreading and osteogenic differentiation depending on cell type (40–90° for hMSCs and ~70° for mMSCs) [46]. However, Ríos-Carrasco et al. [47] found that small wettability differences between hydrophilic shot-blasted (74.7°) and shot-blasted plus acid-etched (64.3°) surfaces had little impact on osseointegration. Therefore, the examined coatings with SBF contact angles of 77–78° fall within the hydrophilic range favourable for bone implants and are likely to support adhesion and proliferation. Nonetheless, factors other than surface wettability, such as roughness or chemical composition, may play a more critical role in their osseointegration performance.

### 3.4. Electrochemical Tests

**Figure 8:**
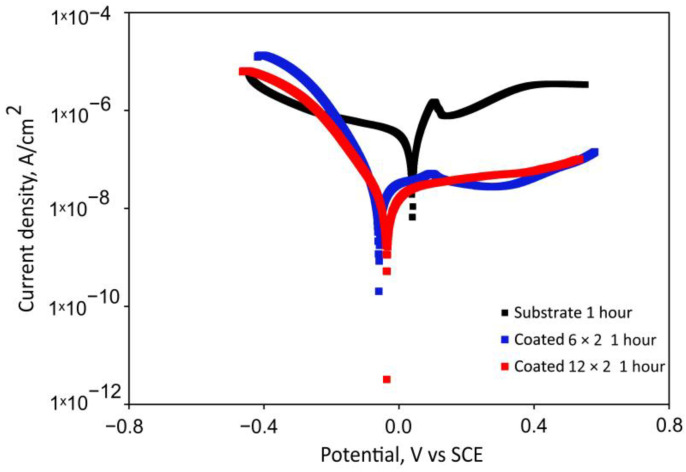
Typical potentiodynamic polarisation curves for the bare substrate and multilayer coatings after 1 h of immersion in SBF, illustrating their comparative corrosion behaviour.

Figure 8 shows the potentiodynamic polarisation curves of the multilayer coatings and the Ti6Al4V substrate following 1 h of immersion in SBF at 37 °C. Corrosion kinetic parameters, such as corrosion potentials (E_corr_) and corrosion current densities (j_corr_), were obtained from the potentiodynamic curves using the Tafel extrapolation method and are summarised in Table 3. Equation (5) was used to evaluate how well the coatings prevented corrosion [48]:

(5) $$P.E.(\%)=\frac{I_{corr-}^{0}I_{corr}^{c}}{I_{corr}^{0}}\times 100\;$$

where I^o^_corr_ and I^c^_corr_ represent the corrosion current densities of the coated and uncoated (bare) samples, respectively.

The corrosion potential E_corr_ of the bare substrate demonstrates higher positive values than the coated specimens (Table 4). The shift toward a more positive electrode potential is indicative of reduced surface reactivity and greater chemical passivity. This behaviour corresponds well with the lower surface roughness quantified for the untreated alloy. However, according to Table 4, both coated samples exhibit a significantly lower corrosion current density (j_corr_) when compared to the bare substrate, indicating a drop in the corrosion rates of these coated samples. Since E_corr_ reflects only the tendency of corrosion initiation, while j_corr_ governs the actual corrosion kinetics, it follows that the coated specimens provide more effective corrosion protection despite their more negative E_corr_. Furthermore, the polarisation curves demonstrate that both coated specimens initiate passivation within the same potential range. Compared to bare Ti6Al4V, coated samples showed lower passivation current density values, indicating enhanced corrosion resistance and reduced metal ion dissolution in SBF during the initial hour of immersion.

**Figure 9:**
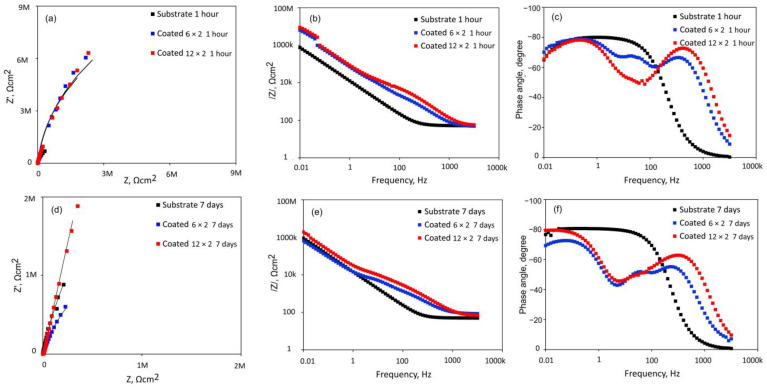
EIS analysis performed on the coated and uncoated specimens following immersion in SBF at 37 °C for periods of 1 h and 7 days: (**a**,**d**) Nyquist diagram; (**b**,**e**) Bode plot; (**c**,**f**) phase angle curve, illustrating the evolution of electrochemical behaviour over time.

Figure 9 presents the Nyquist plots, Bode impedance spectra, and phase angle diagrams obtained from the multilayer coatings and the Ti-6Al-4V substrate, measured at their respective open-circuit potentials after 1 and 168 h of immersion in SBF. All Nyquist diagrams show a flattened capacitive semicircular profile, as seen in Figure 9a. In contrast to both MS coatings, the semicircle for Ti6Al4V under the initial conditions appears more depressed (Figure 9a), indicating that the passive PVD coatings provide better protection than the bare alloy in SBF. With increasing exposure time, however, the impedance arc diameter of the 6 × 2 coating decreases (Figure 9d), whereas that of the 12 × 2 coating slightly increases, suggesting an improvement in its corrosion resistance. After seven days of immersion, the bare alloy exhibits a broader impedance arc and clear capacitive behaviour typical of passive Ti-based alloys. Notably, its semicircle becomes larger than that of the 6 × 2 coating, indicating that the 6 × 2 film undergoes faster corrosion and dissolution than the uncoated substrate over this period.

According to the Bode plot, in the low-frequency domain (1 × 10^−2^∼1 × 10^3^ Hz), the impedance magnitude (|Z|), reflecting the capacitive behaviour of the formed passive films, varies linearly with frequency (Figure 9b,e). Higher |Z| values at 0.01 Hz imply high corrosion resistance. Therefore, the coated samples exhibit the highest overall resistance at first (Figure 9b), but after 7 days of immersion, the bare Ti6Al4V alloy has slightly surpassed the 6 × 2 coated sample (Figure 9e).

Consistent with the behaviour of an insulating oxide layer on a passive surface, the Bode phase angle versus frequency plot for the bare alloy shows a wide plateau, reaching a maximum phase angle of around −80° (Figure 9c,f) [49]. A two-time constant can be seen in the coated samples’ phase angle graphs (Figure 9c,f). Both the low and high-frequency bands exhibited two distinct maximum phase angles corresponding to each time constant. All of this indicates that in the SBF solution, the multilayered coatings exhibit good corrosion resistance. Without compromising the 12 × 2 film’s resistance to corrosion as the immersion period proceeds, the enhanced thickness and sealing of surface discontinuities, as verified by SEM and AFM analysis, improved the passivation film’s stability, leading to greater corrosion resistance than that of the 6 × 2 coatings.

**Figure 10:**
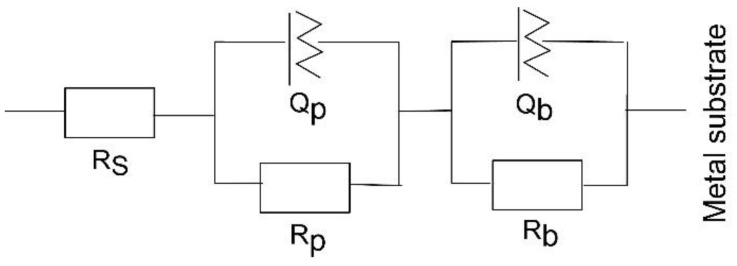
An equivalent circuit used to model the impedance responses of the substrate and coatings at different immersion durations in SBF at 37 °C, representing the electrochemical processes occurring at the interface.

The corrosion behaviour can be quantified by further analysing the EIS spectra through numerical fitting using an equivalent electrical circuit (EEC). EEC model -R_s_(Q_p_R_p_)(Q_b_R_b_)- is employed to model the EIS data (Figure 10). In the sandwich structure with two time constants, the passive film is interpreted as a duplex layer, comprising an outer porous layer and an inner compact layer. In this EEC model, the solution resistance (R_s_) is complemented by the constant phase element (Q_p_) and resistance (R_p_), which correspond to the outer passive layer, while the constant phase element (Q_b_) and resistance (R_b_) are associated with the inner passive layer. To account for surface discontinuities and heterogeneities, a frequency-dependent constant phase element (CPE or Q) with an exponent “n” is used instead of an ideal capacitor to improve the fitting [50]. The exponent “n” indicates the deviation from ideal capacitive or resistive behaviour, with n = 0 corresponding to a pure resistor and n = 1 corresponding to a pure capacitor.

The electrical parameters obtained from fitting the experimental EIS data are presented in Table 5. The chi-square values (χ^2^), representing the sum of squared residuals, are on the order of 10^−3^ to 10^−4^. Consequently, a satisfactory quality of fit is demonstrated by the EEC model that was proposed to fit the experimental data.

According to the fitting results, in contrast to the bare alloy, the R_p_ values for both coatings are noticeably higher than the R_b_ values, suggesting that, initially, the outer barrier layer largely regulates the corrosion resistance of the investigated coated samples. Owing to the higher contact angle with SBF, penetration of the solution into the inner coating layers is expected to be minimal during the initial hours of testing. In contrast, when titanium alloys are immersed in physiological solutions, a duplex oxide structure is typically formed, consisting of an outer porous unsealed TiO_2_ layer and an inner Ti–TiO_2_ layer [51]. For Ti-6Al-4V, the R_b_ values are substantially higher than R_p_, indicating that the compact inner layer of the naturally formed passive film provides greater protection than the outer porous layer. Additionally, the porous oxide film on Ti6Al4V is less uniform and compact compared to the multilayered coatings, as reflected by its lower n_1_ values.

Over the entire test period, the porous layer capacitance (Q_p_) and barrier layer capacitance (Q_b_) of the coated samples exhibited only minor changes (Table 5). After seven days of immersion, the resistance of the compact barrier layer in both coatings increased, while the resistance of the porous layer decreased, suggesting that precipitate formation effectively impeded ion diffusion. The decline in n_1_ values for the PVD films with increasing immersion time indicates that the coating surfaces are more susceptible to electrochemical corrosion. However, the progressive increase in n_2_ values for both coatings suggests that precipitates gradually blocked micropores within the coatings, contributing to their long-term protective behaviour. The 6 × 2 and 12 × 2 coatings demonstrated outer layer resistances 6 × 10^3^ and 1.1 × 10^4^ Ωcm^2^, respectively, that were competitive with the reported outer layer resistance of a CAE-PVD multilayer (24 layers) CrN/TiN coating (2.56 × 10^3^ Ωcm^2^) deposited on the same alloy and immersed for 24 h in Hank’s solution [52]. Critically, both of our coatings exhibited superior inner-layer integrity; specifically, the 12 × 2 film displayed an inner-layer resistance (1.9 × 10^8^ Ωcm^2^) roughly 230 times higher than that of the multilayer CrN/TiN coating (8.23 × 10^5^ Ωcm^2^), highlighting exceptional long-term barrier performance.

**Figure 11:**
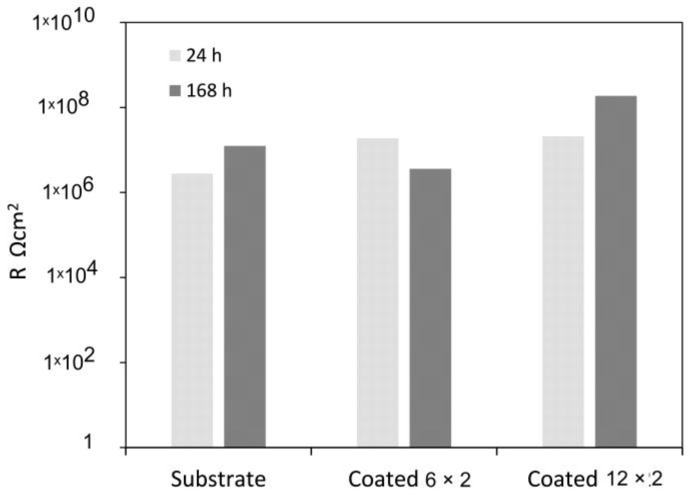
The change in polarisation resistance (R_p_ + R_b_) after 24 and 168 h of immersion in SBF of the uncoated Ti6Al4V and both coatings, illustrating the evolution of corrosion resistance of the coatings over time.

The polarisation resistance values (Figure 11), calculated as the sum of the resistances of the compact barrier layer and the porous passive film (R_p_ + R_b_) [53], initially reach approximately 10^7^ Ωcm^2^ for both coated samples. In general, low coating thickness favours galvanic corrosion and the rapid formation of localised galvanic cells. Accordingly, the reduction in polarisation resistance to about 10^6^ Ωcm^2^ after seven days of immersion (Figure 11) indicates significant degradation of the 6 × 2 coating over prolonged exposure. In contrast, the thicker 12 × 2 coating shows a steady increase in polarisation resistance, reaching approximately 10^8^ Ωcm^2^ after the same immersion period. This behaviour suggests that the 12 × 2 film effectively blocks the penetration of aggressive Cl^−^ ions through surface defects, thereby functioning as a highly efficient anticorrosion layer.

**Figure 12:**
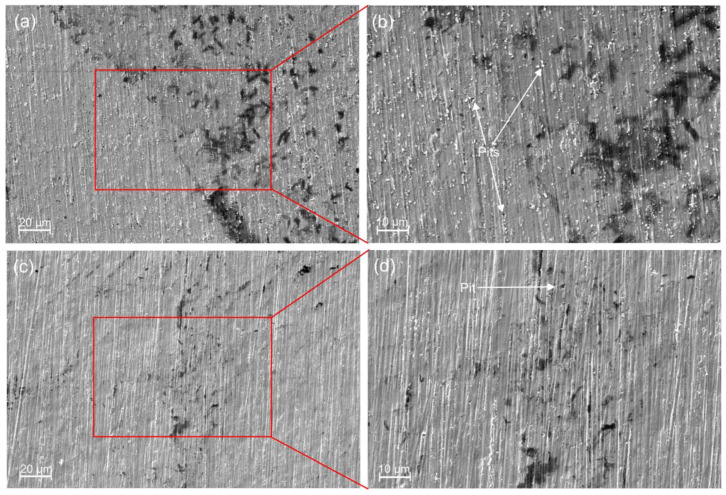
Top surface corrosion morphology (**a**,**b**) 6 × 2 films and (**c**,**d**) 12 × 2 films at different magnifications, showing the distribution and extent of localised corrosion features on the coated surfaces after immersion in SBF. Arrows indicate areas of pitting where applicable.

Post-corrosion SEM analysis revealed no severe surface damage, with only small pit formations observed on both coated samples (Figure 12). In monolayer films, such defects often penetrate through to the underlying substrate. In contrast, for the multilayered coatings, pits were primarily located at the interfaces between layers, with a noticeably higher density in the 6 × 2 coating (Figure 12a,b) compared to the 12 × 2 coating (Figure 12c,d). Importantly, no corrosion sites associated with the alloy substrate were detected in either group, confirming that the corrosion process did not extend to the substrate. This observation is further supported by the high polarisation resistance values recorded during testing, which demonstrate the effective protective role of the multilayer coatings.

### 3.5. Copper Ion Release

**Figure 13:**
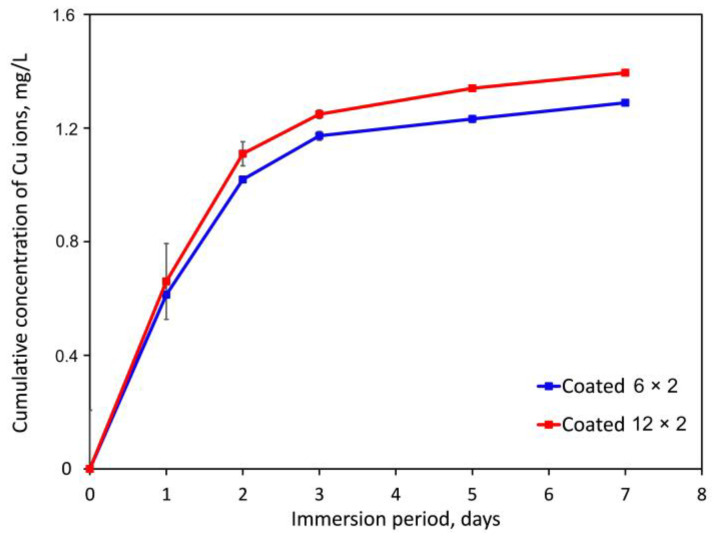
Seven-day cumulative copper ion release from the coated samples into SBF at 37 °C, showing the controlled release behaviour of the multilayer coatings over time. Error bars represent the standard deviation from three independent measurements.

After immersion for 1, 2, 3, 5, and 7 days, we measured the cumulative Cu ion concentration from the coated samples in simulated bodily fluid (SBF) solution at 37 °C using photometric analysis. Figure 13 shows that the 12 × 2 samples with larger Cu contents have a slightly higher concentration of released copper ions. In SBF, Cu-containing coatings displayed comparable Cu release profiles; however, the absolute release for 6 × 2 films was lower. Generally speaking, a substantial copper ion release was seen in the initial hours and persisted for three days, although not in the typical “burst” manner that could cause short-term cytotoxicity [54]. When submerged, Cu ions on the sample surface may gradually dissolve and/or react with the OH^−^ to diffuse into the solution [55]. The Cu-release profiles of the 6 × 2 and 12 × 2 TiO_2_/CuO multilayers exhibit a pronounced faster release in the first 24 h, followed by a rapid exponential-type decay of the incremental release. The release rate decreases by nearly an order of magnitude between day 1 and day 5, which is inconsistent with a diffusion-controlled mechanism (which would exhibit a t^−1^ᐟ^2^ dependence). Instead, the kinetics indicate dissolution-controlled release of CuO located at or near the surface. It is anticipated that this instantaneous burst release will be beneficial for preventing implant-related postoperative infections [56]. It has been proposed that the initial six hours post-implantation constitute a “decisive period” for preventing implant-related infections, offering a critical window to eradicate dormant pathogens near the implant site [57]. After the initial dissolution of accessible CuO nanolayers, the TiO_2_ matrix effectively passivates the surface, resulting in negligible release beyond day 5. The behaviour is consistent across both multilayer architectures, with the 12 × 2 samples exhibiting marginally higher long-term release. It was also reported that TiCu coatings can stimulate the growth of endothelial cells by releasing about 4.5–38.1 µg/L of Cu ions into the PBS solution [58]. However, it was found that DC-MS deposited Ti-Cu films, which released about 30 µg of copper species into Dulbecco’s Modified Eagle Medium over 10 days, resulting in approximately a 50% reduction in MG63 cell viability after just 24 h of incubation [59]. Therefore, throughout the first week following implantation, this release profile might ensure ongoing antibacterial activity and cell stimulation.

### 3.6. Antibacterial Properties

**Figure 14:**
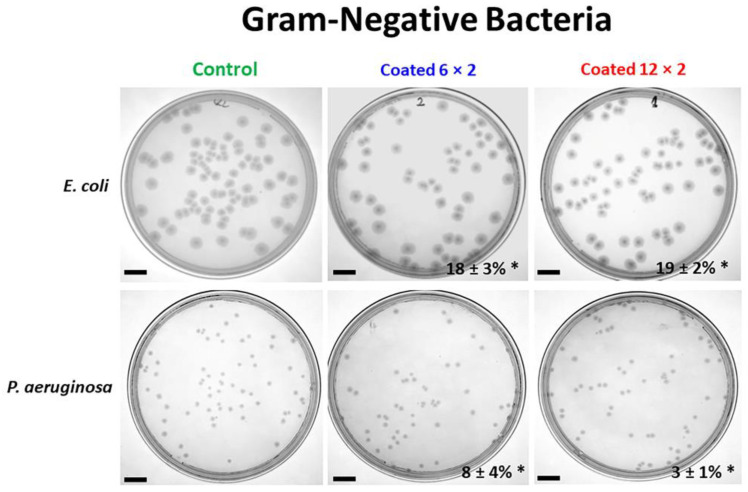
Antibacterial activity of TiO_2_/CuO multilayer coatings against Gram-negative bacteria. Representative CFU plates of *Escherichia coli* and *Pseudomonas aeruginosa* after 24 h incubation on uncoated Ti6Al4V (Control), TiO_2_/CuO 6 × 2 multilayers, and TiO_2_/CuO 12 × 2 multilayers. Both bacterial strains exhibit reduced colony formation on coated surfaces, with *E. coli* showing moderate inhibition (18 ± 3% for 6 × 2 and 19 ± 2% for 12 × 2) and *P. aeruginosa* demonstrating lower but significant inhibition (8 ± 4% for 6 × 2 and 3 ± 1% for 12 × 2) relative to the uncoated substrate. Images shown are representative of *n* = 3 replicates. The data are shown as average ± SD (*n* = 3). Significant deviations from the control sample, Ti6Al4V: * *p* < 0.001. Scale bars = 10 mm.

**Figure 15:**
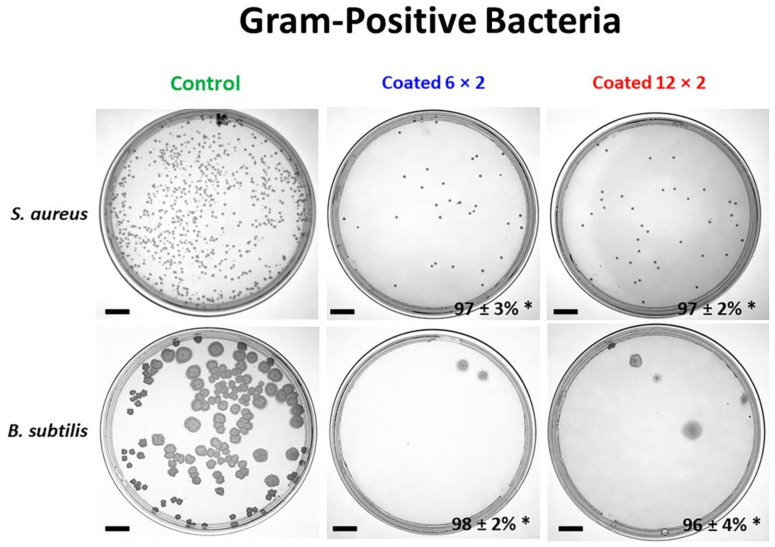
Antibacterial activity of TiO_2_/CuO multilayer coatings against Gram-positive bacteria. Representative CFU plates of *Staphylococcus aureus* and *Bacillus subtilis* after 24 h incubation on uncoated Ti6Al4V (Control), TiO_2_/CuO 6 × 2 multilayers, and TiO_2_/CuO 12 × 2 multilayers. Both Gram-positive species show a pronounced reduction in colony formation on coated surfaces, with growth inhibition values of 97 ± 3% and 97 ± 2% for *S. aureus* on 6 × 2 and 12 × 2 coatings, respectively, and 98 ± 2% and 96 ± 4% for *B. subtilis* compared with the uncoated substrate. Images are representative of *n* = 3 independent experiments. The data are shown as average ± SD (*n* = 3). Significant deviations from the control sample, Ti6Al4V: * *p* < 0.001. Scale bars = 10 mm.

The antibacterial activity of the samples was assessed by comparing four distinct strains of bacteria, two Gram-positive, *S. aureus* and *B. subtilis,* and two Gram-negative, *P. aeruginosa* and *E. coli*, which are important colonisers of implants. The results of antibacterial activity against *P. aeruginosa* and *E. coli* colony formation on the different samples are shown in Figure 14 and Figure 15.

The results presented in Figure 14 and Figure 15 show that Cu-containing TiO_2_ coatings exhibit only a weak antibacterial effect against the Gram-negative bacteria *E. coli* and *P. aeruginosa*. For these strains, the 6 × 2 and 12 × 2 coatings inhibited merely 3% and 8% of *P. aeruginosa* CFUs, and 18% and 19% of *E. coli* CFUs, respectively, compared to the Ti6Al4V control. In contrast, the coatings demonstrated a pronounced antibacterial effect against Gram-positive bacteria, with nearly complete inhibition of bacterial growth. For *S. aureus*, the 6 × 2 and 12 × 2 coatings reduced bacterial growth by 97% relative to the control, while for *B. subtilis*, the inhibition reached 98% and 96% for the 6 × 2 and 12 × 2 coatings, respectively.

Why is the copper emitted from the implants under study more harmful to Gram-positive bacteria than to Gram-negative ones? Gram-negative bacteria have an outer membrane that serves as a first barrier against copper ions. Gram-positives lack this protective layer, allowing copper to reach the cytoplasmic membrane more easily. On the other hand, Gram-negatives can sequester or detoxify Cu^+^ in the periplasm, using systems like CusCFBA and Cu-O efflux pump systems that help export excess copper (mainly Cu(I)) out of the cell. Gram-positives do not have a periplasm, so copper enters the cytoplasm more readily, where it can damage DNA, proteins, and enzymes. Gram-negatives having more complex efflux systems (e.g., CusCFBA) can span the entire envelope and pump copper out, while Gram-positives rely mainly on P-type ATPases like CopA, which only export from cytoplasm to the extracellular space. Both *E. coli* and *P. aeruginosa* possess an outer LPS-rich membrane that restricts Cu^2+^ penetration, reducing ion-mediated antibacterial activity [60,61]. The especially low response of *P. aeruginosa* (3–8%) reflects its strong defence systems, including efficient metal-efflux pumps [62], potent antioxidant enzymes [63], and a highly cross-linked LPS layer [64]. As both multilayer coatings release similar amounts of Cu^2+^, their inhibition values remain comparable, and the marginal effect primarily reflects the biological robustness of Gram-negative bacteria rather than differences in coating architecture.

Another feature of Gram-positive bacteria is the thick peptidoglycan layer, which is rich in negatively charged teichoic acids, which may bind copper ions, increasing local concentrations and enhancing cell toxicity [65]. Similarly, higher bactericidal activity against Gram-positive than against Gram-negative bacteria was reported for other copper-modified titanium surfaces [66,67].

### 3.7. Biofilm Formation

At low concentrations, the Ti-Cu oxide coatings successfully stopped the growth of both Gram-positive and, to some extent, Gram-negative bacteria. Nonetheless, it was not possible to determine if these multilayers could successfully eliminate biofilm formation. Because of the barrier effect of extracellular polysaccharides, the host mechanisms are less effective against biofilm bacteria than against their planktonic counterparts [68,69]. Moreover, biofilm growth on the implant’s surface makes it far more resistant to the action of antimicrobial agents and fluid shear stress. Therefore, it was required to find out if CuO-TiO_2_ multilayered coatings might be used to eliminate the biofilm that the examined bacteria had developed.

**Figure 16:**
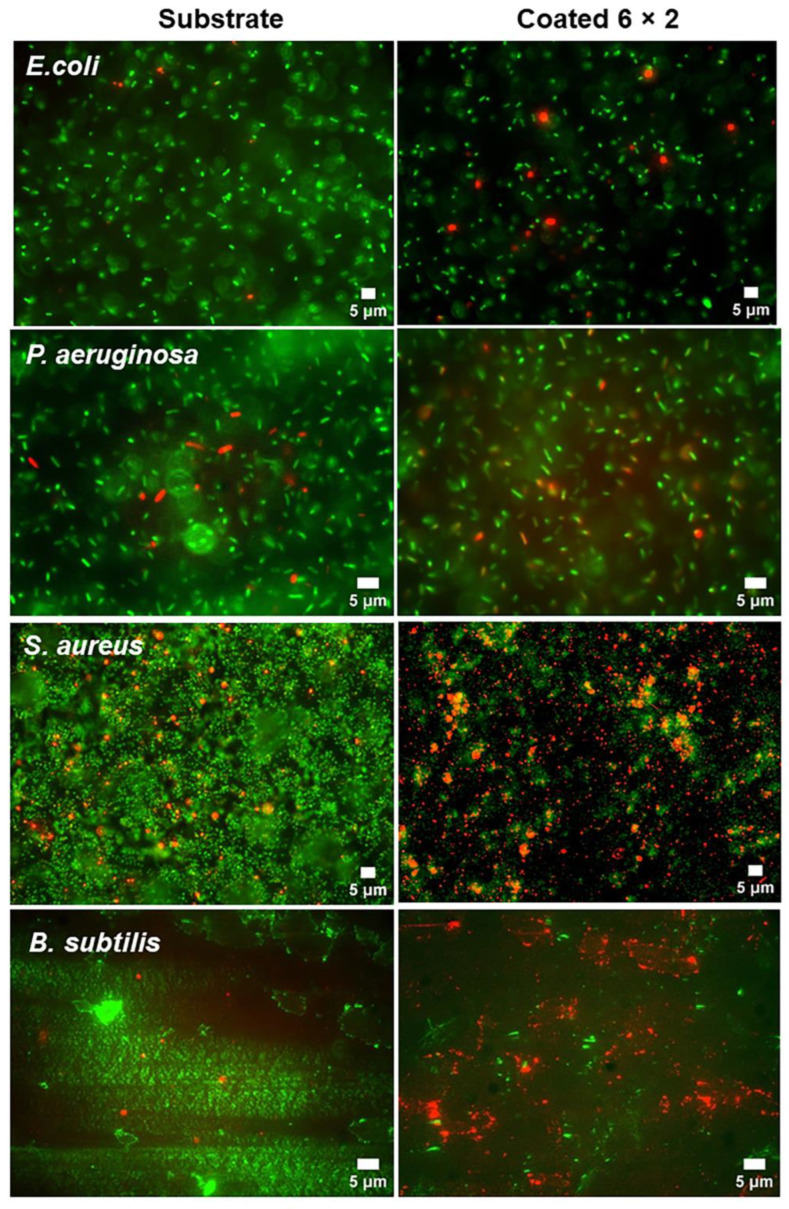
Representative fluorescence micrographs of biofilms formed by *E. coli*, *P. aeruginosa*, *S. aureus*, and *B. subtilis* on uncoated and 6 × 2 coated surfaces. Live cells are shown in green (SYTO9), dead cells in red (propidium iodide). The Ti–Cu oxide coating markedly reduces biofilm coverage and increases bacterial membrane damage across all strains (scale bars: 5 μm).

To evaluate whether the TiO_2_/CuO multilayer coatings inhibit not only planktonic growth but also surface-associated communities, biofilms formed by all four bacterial strains were qualitatively (Figure 16) and quantitatively (Supplementary Table S1) assessed using SYTO 9/propidium iodide (PI) fluorescence imaging. The coatings produced a clear Gram-dependent antibiofilm response, consistent with the CFU plating data. Gram-negative biofilms have moderate susceptibility to the coatings.

For *E. coli*, the multilayer reduced the live biofilm area from 1380.2 to 907.6 µm^2^, corresponding to a 34% decrease in viable biomass, accompanied by a rise in PI-positive area and a drop in viability from 92% to 76% (Supplementary Table S1). *P. aeruginosa* biofilms showed a similar trend: the live area decreased by 14% (2659.2 → 2300.0 µm^2^), with viability decreasing from 96% to 90%. While these changes were less pronounced than in Gram-positive species, they nevertheless demonstrate that the coating imposes measurable physiological stress on surface-attached Gram-negative bacteria, in line with their known copper tolerance mechanisms. In contrast, both Gram-positive strains displayed (Supplementary Table S1) a collapse of biofilm biomass and viability on the coated surfaces. For *S. aureus*, the live biofilm area decreased by approximately 80% (2930.8 → 400.0 µm^2^), with viability dropping sharply from 93% to 18% and a concomitant >9-fold increase in dead-cell staining. *B. subtilis* biofilms were equally affected: viable biomass was reduced by ~85%, and the dead-area signal increased from 108.0 to 7780.6 µm^2^, indicating extensive membrane disruption and biofilm destruction. These strong antibiofilm effects align with the exceptionally high CFU inhibition observed for Gram-positive species (Figure 15) and reflect their intrinsic vulnerability to copper ions due to the absence of an outer membrane and limited capacity for Cu detoxification.

The antibacterial performance of the TiO_2_/CuO multilayers is primarily governed by Cu^+^/Cu^2+^ ion release, which is known to damage bacterial membranes, proteins, and DNA [70]. This is supported by our fluorescence images showing widespread PI-positive, membrane-compromised bacteria. Sustained copper release from similar CuO/TiO_2_ systems has also been reported to suppress biofilm formation [71]. A secondary contribution arises from direct contact killing, as surface-exposed copper can induce membrane rupture and protein denaturation [72,73,74]. A third, less dominant mechanism may involve CuO–TiO_2_ redox interactions, where Cu^+^/Cu^2+^ cycling and ROS generation further enhance antibacterial activity [71].

Overall, the biofilm analysis confirms that TiO_2_/CuO multilayers exert substantial bactericidal pressure on Gram-positive biofilms and moderate inhibitory effects on Gram-negative biofilms, mirroring the trends observed in planktonic viability. Copper’s antibacterial effect relies on free Cu^+^ and Cu^2+^ ions, which quickly bind to proteins and other molecules in the body, reducing their activity over time [75,76,77,78]. CuO/TiO_2_ hybrid coatings reduce protein buildup by creating a more hydrophilic, negatively charged surface that repels proteins [71,79]. This allows for a steady release of copper ions, preventing blockage and inactivation. The sustained release creates low-level stress that stops bacteria from attaching and forming biofilms [71,79]. As a result, CuO/TiO_2_ coatings keep their antibacterial effect longer than pure CuO, especially in protein-rich environments [76,77,78,79]. Importantly, the coatings not only reduce biofilm mass but also significantly increase the proportion of nonviable cells, demonstrating that copper-driven membrane damage remains an active mechanism at the biofilm–surface interface. These results establish the TiO_2_/CuO multilayer system as a promising surface modification for preventing bacterial colonisation on biomedical titanium substrates, particularly against clinically relevant Gram-positive pathogens.

### 3.8. In Vitro Viability Tests with the MG-63 Cell Line

**Figure 17:**
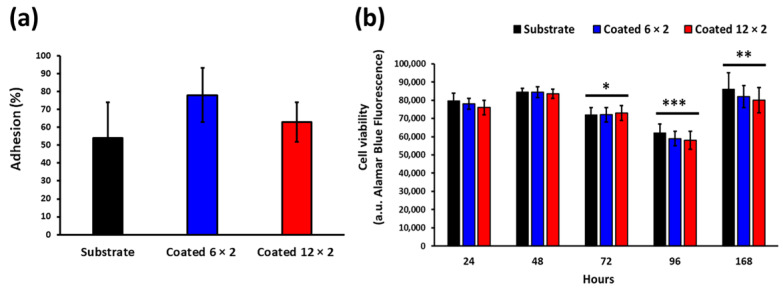
Cell adhesion and metabolic activity of MG-63 cells cultured on uncoated and Ti–Cu oxide–coated Ti6Al4V surfaces. (**a**) Percentage of initially attached cells after 3 h expressed relative to the total number of seeded cells. No significant difference among surfaces was detected (one-way ANOVA, Bonferroni *p* > 0.05). (**b**) Cell viability determined by AlamarBlue assay (mean ± SD, *n* = 9) for substrate (black), coated 6 × 2 (blue), and coated 12 × 2 (red) samples measured over 7 days. * *p* < 0.05, ** *p* < 0.01, *** *p* < 0.001 versus 24 h within each sample (Bonferroni-adjusted).

The first step was to investigate how the coated surfaces affect the MG-63 cells’ attachment. Initial adhesion was not affected by surface type (Figure 17a). A one-way ANOVA comparing the percentage of cells retained 3h after culturing across substrates, 6 × 2, and 12 × 2 coated samples showed no main effect of surface (*p* > 0.05). Bonferroni-adjusted pairwise comparisons were non-significant for all contrasts (all *p* > 0.05). Thus, within the precision of this experiment, the Ti–Cu oxide multilayers do not reduce early cell attachment relative to the uncoated substrate.

The next step was to investigate how the coated surfaces affect the viability of MG-63 cells. AlamarBlue fluorescence revealed a time-dependent variation in cell viability on both coated and uncoated specimens (Figure 17b, Table S2). During the first 48 h, all groups—substrate (Ti6Al4V), 6 × 2, and 12 × 2 coated samples showed comparable metabolic activity (*p* > 0.05). At 72 h, a modest but significant reduction in viability was detected across all samples (* *p* < 0.05, Table S2), which became most pronounced at 96 h (** *p* < 0.001, Table S1), indicating a transient metabolic decline likely associated with cell confluence adaptation or nutrient depletion. By 168 h, fluorescence values recovered significantly (*** *p* < 0.01, Table S2) and approached or exceeded early-time levels, suggesting re-establishment of a stable proliferative state. No significant differences were observed among the three surface types at any time point, demonstrating that the copper coatings did not exert cytotoxic effects and supported sustained metabolic activity comparable to the uncoated substrate.

Mauchly’s test indicated that the assumption of sphericity was violated for the within-subjects factor Time (χ^2^(9) = 34.25, *p* < 0.001). Therefore, Greenhouse–Geisser corrections (ε = 0.61) were applied. The repeated-measures ANOVA revealed a highly significant main effect of Time (F(2.43, 58.34) = 100.26, *p* < 0.001, Table S2), demonstrating that Alamar Blue fluorescence and, thus, cell viability changed during incubation. Trend analyses showed that the temporal variation followed a pronounced curvilinear pattern, with significant linear (F(1, 24) = 8.31, *p* = 0.008, Table S3), quadratic (F(1, 24) = 108.13, *p* < 0.001, Table S3), and cubic (F(1, 24) = 729.24, *p* < 0.001) components, indicating an initial decrease in viability followed by recovery at later time points. The overall Time × Sample interaction was not significant (F(4.86, 58.34) = 1.57, *p* = 0.19, Table S3), suggesting that the three surfaces (substrate, 6 × 2, and 12 × 2 coated) exhibited similar time-dependent profiles. However, a significant quadratic Time × Sample interaction (F(2, 24) = 4.59, *p* = 0.021, Table S2) indicated minor differences in the curvature of these responses. The between-subjects effect of Sample was significant (F(2, 24) = 5.17, *p* = 0.014, Table S3), confirming that the average viability level differed among the three surfaces. Altogether, these findings show that while all samples displayed comparable temporal behaviour, their overall metabolic activity differed, with viability varying non-linearly throughout the seven-day culture period.

The transient decline in Alamar Blue metabolic activity observed in MG-63 cells at 72–96 h (Figure 17b) is consistent with a combination of culture-related factors and a short-lived cellular adaptation to early Cu exposure from the coated surfaces. MG-63 cells proliferate rapidly and approach confluence, which reduces mitochondrial turnover and metabolic enzyme activity due to contact inhibition. This alone can lower the Alamar Blue signal independently of cytotoxicity. In addition, prolonged incubation in the medium renewal leads to nutrient depletion and pH drift, further decreasing metabolic output at these intermediate time points. During this short adaptation period, intracellular redox balance and mitochondrial activity may be transiently affected, contributing to the temporary reduction in Alamar Blue signal at 72–96 h. The fact that metabolic activity fully recovers by 168 h across all surfaces, together with the marked increase in MT, suggests that MG-63 cells successfully adapt to the initial Cu microenvironment. Thus, the observed dip in viability reflects a reversible metabolic adaptation phase rather than material-induced cytotoxicity.

The next step was to examine the effect of coated samples on cell morphology and F-actin distribution.

### 3.9. Cell Morphology

**Figure 18:**
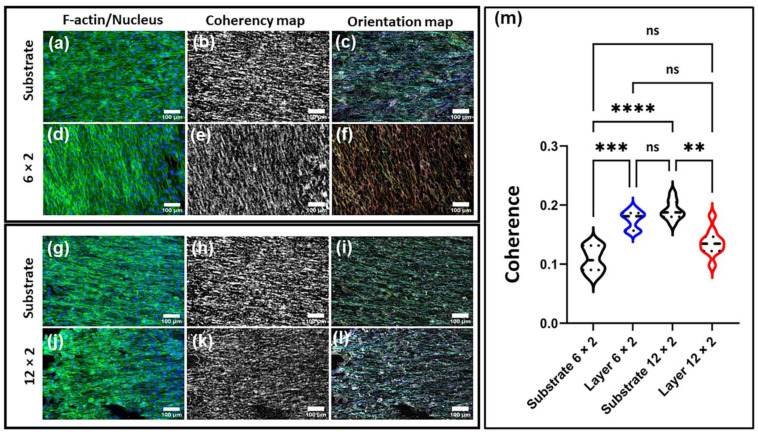
F-actin alignment and OrientationJ analysis of cells cultured on TiOxNy-based substrates and multilayer coatings. (**a**–**c**) Substrate 6 × 2; (**d**–**f**) Layer 6 × 2; (**g**–**i**) Substrate 12 × 2; (**j**–**l**) Layer 12 × 2. Representative fluorescence images of F-actin (green) and nuclei (blue) are shown in panels (**a**,**d**,**g**,**j**). Coherency maps generated with OrientationJ illustrate the degree of local cytoskeletal alignment (**b**,**e**,**h**,**k**). Orientation maps (**c**,**f**,**i**,**l**) visualise fibre direction using a fixed hue–saturation colour LUT. (**m**) Violin plots of OrientationJ coherency values obtained from ten ROIs per image. Horizontal lines indicate median and interquartile range; dots represent individual ROI measurements. Statistical analysis was performed using the Kruskal–Wallis test with Dunn’s post hoc correction. **** *p* < 0.0001, *** *p* < 0.001, ** *p* < 0.01, ns: not significant. Scale bars: 100 µm.

Figure 18 reveals distinct nanotopographical differences between the copper-containing coatings. The 6 × 2 films exhibited finely oriented, shallow linear features and a thinner architecture (~186 nm), whereas the 12 × 2 coatings were thicker (~341 nm) with a denser, more columnar and less directionally defined surface. Consistent with these morphologies, F-actin staining (Figure 18a,d,g,j) showed that MG-63 cells on 6 × 2 films adopted elongated morphologies with long, parallel ventral stress fibres and high field-wide alignment. Cells cultured on both multilayer coatings displayed visibly enhanced cytoskeletal organization compared with their respective substrates. In the representative fluorescence images, actin fibres on the 6 × 2 and 12 × 2 multilayers appeared more uniformly aligned along a common axis, whereas cells on the corresponding substrates exhibited a more disordered, multidirectional actin architecture. These qualitative differences were corroborated by OrientationJ coherency and orientation maps, which demonstrated higher local alignment and a narrower angular distribution on the multilayer coatings (Figure 18, panels d–f and j–l) relative to their substrate controls (panels a–c and g–i).

Quantitative analysis of coherency values extracted from ten ROIs per image demonstrated statistically significant increases in cytoskeletal alignment on both multilayer coatings (Figure 18m). For the 6 × 2 system, the multilayer coating showed markedly higher coherency than the substrate (*p* < 0.001), indicating a pronounced contact-guidance effect imparted by the multilayer architecture. A similar trend was observed for the 12 × 2 system, where the multilayer also supported significantly greater alignment than its substrate (*p* < 0.01). Comparisons between substrates further revealed that the 12 × 2 substrate exhibited slightly higher alignment than the 6 × 2 substrate (*p* < 0.0001), while the two multilayer coatings displayed no significant difference. Collectively, these data show that multilayered TiOxNy coatings consistently promote stronger actin alignment than the underlying substrates.

Cytoskeletal coherency is a key upstream regulator of intracellular tension and focal-adhesion maturation, and is tightly coupled to activation of major mechanosensitive pathways. Enhanced actin alignment stabilises integrin–adhesion complexes and facilitates directional force transmission [80,81], promoting RhoA/ROCK-dependent actomyosin contractility [82]. Increased intracellular tension generated along aligned stress fibres is a well-established trigger of YAP/TAZ nuclear translocation [83,84], and integrin–cytoskeleton mechanocoupling [85]. Mechanical deformation of cell–cell and cell–matrix junctions also regulates β-catenin activation [86]. Therefore, the higher coherence observed on the multilayer coatings suggests more efficient mechanotransductive signalling, consistent with enhanced focal-adhesion maturation, anisotropic force propagation, and downstream transcriptional responses associated with cell spreading, phenotype stabilisation, and material integration.

The finding that both multilayer coatings induced similar levels of alignment despite differences in film thickness and nanoscale architecture implies that the presence of the multilayer structure itself—rather than incremental adjustments in layer configuration—is the dominant factor generating directional guidance cues. Conversely, the lower alignment observed on the substrates indicates a less instructive interface, where cells experience more isotropic mechanical input and therefore adopt less organised cytoskeletal patterns. Contact guidance arises when ridge–groove anisotropy biases focal-adhesion nucleation and actomyosin bundling along a dominant axis. The shallow, linearly oriented nanotexture of the 6 × 2 coating likely reduces lateral curvature and stabilises aligned adhesions, enabling robust stress-fibre formation and higher anisotropy. In contrast, the greater thickness and columnar texture of the 12 × 2 surface likely increase nanoscale roughness and slope variability, partially diluting directional cues and producing more locally reoriented fibres. Both coated surfaces, however, promote elongated, aligned morphologies with substantial stress-fibre formation compared with the uncoated substrates. These cytoskeletal differences occur without any detectable metabolic penalty (Figure 17), indicating that the coatings are both biocompatible and guidance-competent.

### 3.10. Osteoblastic Differentiation

**Figure 19:**
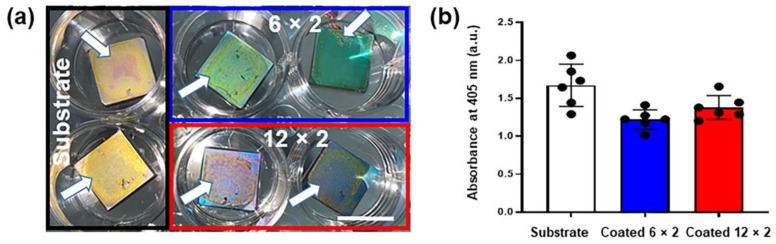
Visual assessment of coated samples and quantification of osteogenic mineralisation. (**a**) Representative photographs of uncoated substrates and copper-containing multilayer coatings (6 × 2 and 12 × 2). Distinct interference colours reflect differences in film architecture and thickness. White arrows indicate characteristic surface features. Scale bar: 5 mm. (**b**) Alizarin Red S quantification (absorbance at 405 nm) after osteogenic induction. Both 6 × 2 and 12 × 2 coatings supported mineral matrix formation comparable to the uncoated substrate (*p* > 0.05). Data are presented as mean ± SD with individual data points (*n* = 6). Bar 14 mm.

After 21 days of osteoblastic differentiation, Alizarin Red S staining revealed pronounced calcium deposition in MG-63 cultures on both coated and uncoated samples (Figure 19). Visual inspection (Figure 19a) showed similar red-orange mineral nodules on all surfaces, confirming active extracellular matrix mineralisation. Quantitative dye extraction (Figure 19b) demonstrated that cells on copper-containing (6 × 2 and 12 × 2) surfaces produced slightly lower absorbance values at 405 nm compared with the uncoated substrate, although differences were not statistically significant (*p* > 0.05). These findings indicate that both coatings support osteogenic differentiation and mineral matrix formation to a degree comparable with the native titanium substrate.

The retention of strong Alizarin Red S signals on the coated samples confirms that coated films preserve the osteogenic potential of MG-63 cells over extended culture. The minor decrease in absorbance on the 6 × 2 and 12 × 2 coatings likely reflects subtle differences in surface chemistry and film thickness, which can influence local calcium phosphate nucleation rather than impairing cell differentiation. Even with similar wettability and identical top CuO composition, the difference in total thickness (and thus defect structure and electrochemical reactivity) may control the density and kinetics of calcium phosphate nucleation. The combination of steady ionic stimulation and increased stiffness can synergistically promote osteogenic differentiation, protein adsorption favourable for mineral nucleation, and the formation of Cu-rich surface species that act as nucleation centres for calcium phosphate. Nevertheless, the absence of significant differences indicates that both coatings maintain an environment conducive to osteoblast maturation and mineral deposition, confirming their cytocompatibility and osteoconductivity.

### 3.11. Metallothionein Response

**Figure 20:**
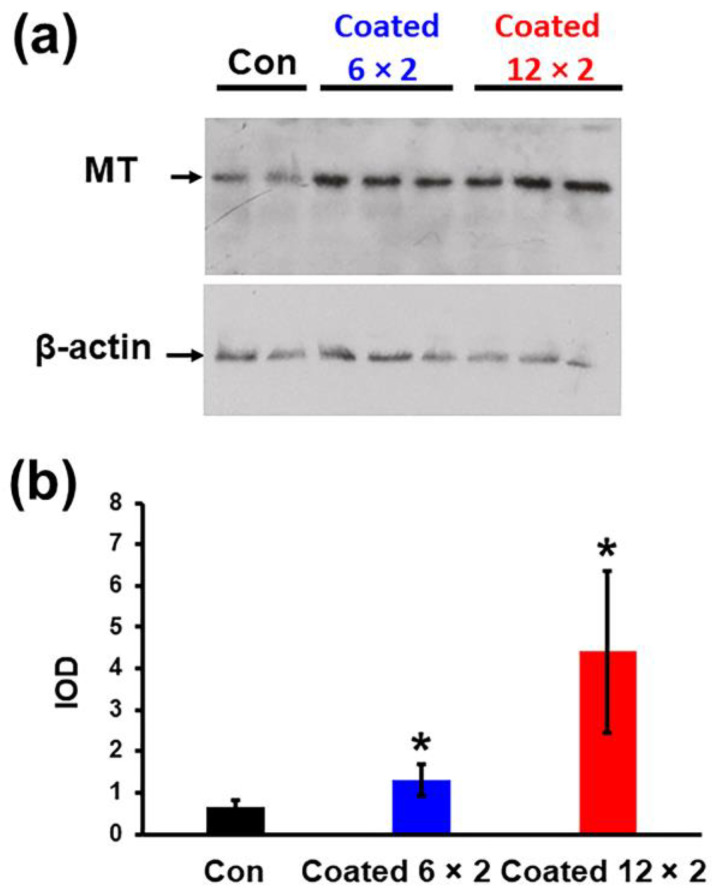
Representative Western blot analysis of metallothionein (MT) expression in MG-63 cells cultured for 24 h on uncoated substrates (Con) and on 6 × 2 and 12 × 2 coated surfaces (**a**). β-actin was used as a loading control. Panel (**b**) shows the integrated optical density (IOD) of MT normalised to β-actin. Data are presented as mean ± SD. * *p* < 0.05 compared with the control.

Metallothioneins (MT) are cysteine-rich metal-binding proteins that regulate redox balance and metal homeostasis. The elevated MT levels (Figure 20) observed here likely result from Cu^2+^ ion release from the 6 × 2 and 12 × 2 oxide layers (Figure 13), leading to mild intracellular signalling that stimulates antioxidant and cytoprotective pathways. This adaptive upregulation enhances cellular tolerance to metal ions and may contribute to the sustained viability and differentiation seen in Alamar Blue and Alizarin Red S results. The stronger MT response on the 12 × 2 surface corresponds with its higher Cu content and slightly greater ion release (Figure 13), confirming a dose-dependent cellular adaptation rather than toxicity. Cells on both coated samples—particularly 12 × 2—exhibited a marked upregulation of MT protein compared to control cells grown on uncoated titanium (*p* < 0.05). The expression increased progressively with coating thickness and Cu content (Control < 6 × 2 < 12 × 2). This finding indicates that the coatings could induce moderate oxidative or ionic microstimulation that activates cellular defence mechanisms without causing cytotoxic stress (Figure 17).

The biological response of cells to the Cu-containing multilayers reflects a finely tuned interplay between the coatings’ physicochemical characteristics and their ionic activity. The Alamar Blue assay (Figure 17) demonstrated sustained viability on both 6 × 2 and 12 × 2 multilayer coatings, confirming that the incorporated Cu levels do not elicit cytotoxicity. The transient decrease in metabolic activity at 72–96 h, followed by recovery at 168 h, likely represents an adaptive metabolic adjustment during early attachment, spreading, and reorganisation on the modified surfaces. Fluorescence imaging of F-actin (Figure 18) further revealed that osteoblastic cells exhibited pronounced cytoskeletal alignment on the 6 × 2 coatings, whereas the thicker 12 × 2 films (~341 nm), characterised by a denser and less anisotropic nanotopography, supported reduced cytoskeletal coherence. These findings indicate that nanoscale anisotropy—rather than roughness magnitude alone—governs contact guidance and collective cell orientation. Concurrently, Cu^2+^ release from the coatings activated endogenous antioxidant and metal-binding pathways without impairing cell metabolism. The observed metallothionein (MT) induction represents a cytoprotective response that enhances redox buffering and tolerance to metal ions. Collectively, these results show that the multilayer Ti–Cu oxide coatings integrate mechanical stability and corrosion resistance with favourable biological performance, promoting cellular adaptation, cytoskeletal organisation, and osteogenic maturation—key attributes for multifunctional implant coatings.

The modest reduction in Alizarin Red S staining observed on the coated samples may reflect subtle, yet functionally relevant, differences in surface chemistry and ion-release behaviour between the multilayer Ti–Cu oxide films and the uncoated Ti6Al4V substrate. The deposition of the 6 × 2 and 12 × 2 films can modify critical physicochemical parameters—including surface charge, oxide composition, roughness, and hydrophilicity—which are known regulators of integrin-mediated adhesion and early osteogenic signalling. Such changes may slightly influence the initial polarity and mechanotransduction state of MG-63 cells, thereby affecting subsequent mineral deposition.

Film thickness and nanostructural architecture also appear to be contributory. The thicker 12 × 2 multilayer may dampen or reorganise nanoscale cues that normally promote osteoblast maturation, whereas the thinner 6 × 2 films may preserve more of the underlying topographical guidance. Even subtle variations in these architectural features can influence the efficiency with which pre-osteoblasts transition from a proliferative state to matrix mineralisation. Cu ion release kinetics represent another plausible factor. Although Cu at low concentrations can stimulate osteogenic signalling, transient elevations during the early adaptation phase may activate redox-responsive pathways, including MT upregulation (Figure 20). Such early adaptive responses, while not cytotoxic, may temporarily delay or slightly reduce mineral deposition, consistent with the modest decrease in Alizarin Red S absorbance (Figure 19). Importantly, metabolic activity and viability remained comparable across all groups at later time points, indicating that these effects reflect fine modulation of osteoblast maturation rather than impaired viability.

Taken together, the small reduction in mineralisation on the coated samples likely arises from the combined influence of modified surface chemistry, altered nanoscale architecture, and early Cu ion-driven adaptive responses. These effects are subtle and consistent with a scenario in which the coatings modulate, rather than inhibit, osteogenic differentiation.

## 4. Conclusions

In this study, multilayer CuO/TiO_2_ coatings with 6 × 2 and 12 × 2 architectures were deposited on Ti6Al4V by magnetron sputtering and evaluated for structural, electrochemical, biological, and antibacterial performance. The multilayer design enabled controlled Cu distribution and ion release, influencing corrosion resistance, wettability, and cellular response. XRD confirmed anatase and rutile TiO_2_ with monoclinic CuO, while the architecture ensured uniform copper distribution. Coating thickness increased from ~180 nm (6 × 2) to ~340 nm (12 × 2), although AFM showed similar surface roughness (~50 nm), higher than that of the bare substrate (~20 nm). Both coatings improved hardness and corrosion protection, with the 12 × 2 structure achieving the highest polarisation resistance (~10^8^ Ωcm^2^) after seven days in SBF. Antibacterial tests showed moderate inhibition of Gram-negative strains but >96% suppression of Gram-positive bacteria. MG-63 assays demonstrated good viability, osteogenic differentiation, and no cytotoxicity. Morphological and OrientationJ analyses indicated enhanced cytoskeletal alignment and mechanotransduction, supporting stronger osteoblastic adhesion and function. Upregulated metallothionein expression further reflected a favourable response to controlled Cu release. Overall, the multilayer Ti–Cu oxide coatings provide combined antimicrobial activity and cytocompatibility, highlighting their potential for multifunctional implant surfaces. Future work will examine long-term degradation and ion-release behaviour under physiological conditions.

## Figures and Tables

**Table 1:** Copper, titanium, and oxygen content on the surface of the multilayered coatings.

Element	6 × 2 Coating		12 × 2 Coating	
	wt%	Sigma	wt%	Sigma
Ti	41.79	0.66	30.12	0.68
Cu	29.83	0.59	40.10	0.67
O	28.38	0.6	29.78	0.6
Total	100	-	100	-

**Table 2:** Surface roughness values for 6 × 2 and 12 × 2 coatings and substrates.

Sample	S_a_, nm	S_z_, µm	S_sk_
Substrate	20.96 ± 6.06	0.18 ± 0.07	0.18 ± 0.06
Coated 6 × 2	45.10 ± 14.34	0.34 ± 0.14	1.03 ± 0.15
Coated 12 × 2	49.17 ± 16.04	0.37 ± 0.15	0.30 ± 0.07

**Table 3:** Microhardness, critical adhesion strength, and friction coefficient measurements (with ±standard deviation) of the substrate and coated samples.

Sample	HK_0.005_ (kgf mm^−2^)	F_C_ (N)	COF
Substrate	866.5 ± 68	-	0.36 ± 0.02
Coated 6 × 2	1429 ± 79	9.2 ± 0.2	0.19 ± 0.01
Coated 12 × 2	2434 ± 93	14.8 ± 0.5	0.26 ± 0.01

**Table 4:** Values of corrosion potential (E_corr_), corrosion current density (j_corr_), and protection efficiency (P.E.) determined for both the substrate and coated samples after 1 h of immersion.

Sample	E_corr_ (mV vs. SCE)	j_corr_ (10^−9^ A cm^−2^)	P.E. (%)
Ti6Al4V	39.6	434	-
Coated 6 × 2	−59.8	34.6	92
Coated 12 × 2	−36.7	24.9	94.3

**Table 5:** Electrochemical parameters obtained through numerical fitting for the uncoated and the oxide-coated samples.

Sample Time	Ti6Al4V		Coated 6 × 2		Coated 12 × 2	
	24 h	168 h	24 h	168 h	24 h	168 h
Q_p_, (Ω^−1^cm^−2^s^n^)	2.1 × 10^−3^	7.3 × 10^−7^	2.5 × 10^−6^	8.3 × 10^−6^	2 × 10^−6^	2.9 × 10^−6^
n_1_	0.12	0.57	0.95	0.71	0.94	0.75
R_p_, (Ωcm^2^)	17.8	17.4	1.9 × 10^7^	6 × 10^3^	2.1 × 10^7^	1.1 × 10^4^
Q_b_, (Ω^−1^cm^−2^s^n^)	1.5 × 10^−5^	1.3 × 10^−5^	5.3 × 10^−6^	1.7 × 10^−5^	7.5 × 10^−7^	6.9 × 10^−6^
n_2_	0.9	0.9	0.74	0.87	0.85	0.9
R_b_, (Ωcm^2^)	2.8 × 10^6^	1.3 × 10^7^	7.9 × 10^3^	3.6 × 10^6^	9.2 × 10^3^	1.9 × 10^8^

## Data Availability

The original contributions presented in this study are included in the article/Supplementary Materials. Further inquiries can be directed to the corresponding authors.

## Supplementary Materials

The following supporting information can be downloaded at: https://www.mdpi.com/article/10.3390/jfb17010016/s1. Table S1. SYTO9/PI-based quantitative biofilm metrics (live biomass, dead biomass, total biofilm area, viability, and inhibition percentage) measured after 24 h incubation on control and TiO_2_/CuO (6 × 2) multilayer-coated Ti6Al4V substrates; Table S2. Descriptive statistics of Alamar Blue fluorescence intensity (mean ± SD, n = 9) for MG-63 cells cultured on Substrate, Coated 6 × 2, and Coated 12 × 2 samples at 24–168 h; Table S3. Summary of statistical analyses for Alamar Blue cell-viability assay.
