# Supplementary material for: Multilayer Ti–Cu Oxide Coatings on Ti6Al4V: Balancing Antibacterial Activity, Mechanical Strength, Corrosion Resistance, and Cytocompatibility
Source: J Funct Biomater. 2025 Dec 26;17(1):16. doi: 10.3390/jfb17010016 (PMC12842340; doi:10.3390/jfb17010016)
Supplement: Supplementary file 1 [file jfb-17-00016-s001.zip › jfb-4008906-supplementary.pdf]

OrientationJ Workflow, Step-by-Step Protocol

1. Open F-actin fluorescence images in Fiji.

2. Convert images to 8-bit and apply background subtraction (rolling ball radius: 50 px).

3. Run OrientationJ Analysis using cubic-spline gradients and a window radius of 2 px.

4. Generate the orientation map and coherency map.

5. Select ten non-overlapping ROIs of identical size and extract coherency values using OrientationJ Measure.

6. Export coherency values for statistical analysis (SPSS).

Supplementary Tables

Table S1. SYTO9/PI-based quantitative biofilm metrics (live biomass, dead biomass, total biofilm area, viability, and inhibition percentage) measured after 24 h incubation on control and TiO_2/CuO (6×2) multilayer-coated Ti6Al4V substrates. *p<0.05, **p<0.001 compare to 24h

Strain	Live area μm^2	Dead area μm^2	Total Area μm^2	Viability %	Inhibition of live biofilm%
E.coli control	1380.2	116.8	1497	92% ± 5%	34
E.coli sample (6x2)	907.6*	279*	1186.6*	76% ± 2.5%*	
P. aeruginosa control	2659.2	110	2769.6	96% ± 1.1%	14
P. aeruginosa sample (6x2)	2300*	232*	2532*	90% ± 1.2%*	
S. aureus control	2930.8	205.4	3136.2	93% ± 2.3%	80
S. aureus sample (6x2)	400**	1860**	2260**	18% ± 4.6%**	
B. subtilis control	1771.2	108	1879.2	94% ± 1.2%	85
B. subtilis sample (6x2)	265.6**	7780.6**	1046.2**	25% ± 3.9%**	

Table S2. Descriptive statistics of Alamar Blue fluorescence intensity (mean ± SD, n = 9) for MG-63 cells cultured on Substrate, Coated 6x2, and Coated 12x2 samples at 24–168 h. *p<0.05, **p<0.01, ***p<0.001 compare to 24h.

Time (h)	Substrate (Mean ± SD)	Coated 6x2 (Mean ± SD)	Coated 12x2 (Mean ± SD)
24	79800 ± 4000	78000 ± 3000	76000 ± 4000
48	84500 ± 2000	84500 ± 3000	83500 ± 2500
72	72000 ± 4000*	72000 ± 4000*	73000 ± 4000*
96	62000 ± 5000***	59000 ± 4000***	58000 ± 5000***

168	86000 ± 9000**	82000 ± 6000**	80000 ± 7000**

Table S3. Summary of statistical analyses for Alamar Blue cell-viability assay

Effect	Statistical test	df	F (or χ^2)	p-value	Interpretation
Time	Repeated-measures ANOVA (Greenhouse–Geisser corrected)	2.43, 58.34	100.26	< 0.001	Viability changes significantly over time
Sample	Between-subjects ANOVA (mixed design)	2, 24	5.17	0.014	Overall viability differs among all samples
Time × Sample	Repeated-measures ANOVA (Greenhouse–Geisser corrected)	4.86, 58.34	1.57	0.185	Not significant; similar time-course for all samples
Linear trend (Time)	Within-subjects contrast	1, 24	8.31	0.008	Significant linear component
Quadratic trend (Time)	Within-subjects contrast	1, 24	108.13	< 0.001	Pronounced curvature in the viability pattern
Cubic trend (Time)	Within-subjects contrast	1, 24	729.24	< 0.001	Strong complex curvature
Quadratic Time × Sample	Within-subjects contrast	2, 24	4.59	0.021	Minor differences in curvature

between samples

Repeated-measures ANOVA with Greenhouse–Geisser correction was applied for within-subject effects (Time, Time × Sample). Post-hoc pairwise comparisons were adjusted using the Bonferroni procedure.
